# A novel bis-triazole scaffold accessed via two tandem [3 + 2] cycloaddition events including an uncatalyzed, room temperature azide–alkyne click reaction

**DOI:** 10.3762/bjoc.18.175

**Published:** 2022-12-02

**Authors:** Ksenia Malkova, Andrey Bubyrev, Vasilisa Krivovicheva, Dmitry Dar’in, Alexander Bunev, Mikhail Krasavin

**Affiliations:** 1 Saint Petersburg State University, Saint Petersburg 199034, Russian Federation; 2 Medicinal Chemistry Center, Togliatti State University,445020 Togliatti, Russian Federation; 3 Immanuel Kant Baltic Federal University, Kaliningrad 236016, Russian Federation

**Keywords:** α-acetyl-α-diazomethane sulfonamide, intramolecular click reaction, uncatalyzed, room temperature, 1,2,3-triazoles

## Abstract

The previously described α-acetyl-α-diazomethanesulfonamide was employed in a three-component reaction with azide-containing benzaldehydes and propargylamines. Besides the initial formation of the triazole core, the reaction proceeded further, in uncatalyzed fashion at room temperature and yielded, after intramolecular azide–alkyne click reaction novel, structurally intriguing bistriazoles.

## Introduction

1,2,3-Triazoles are well-established heterocycles in drug discovery [1] and are even considered pharmacophores (i.e., structural motifs defining the compound’s biological activity profile) on their own [2]. Therefore, synthetic methods allowing to construct a 1,2,3-triazole heterocycle are a valuable part of the drug discovery chemistry toolbox. For the same reason, development of new methods [3] to either build 1,2,3-triazoles de novo and/or incorporate them into polycyclic scaffolds is a worthy undertaking which can help discover biological activity associated with hitherto unattainable scaffolds.

**Figure 1:**
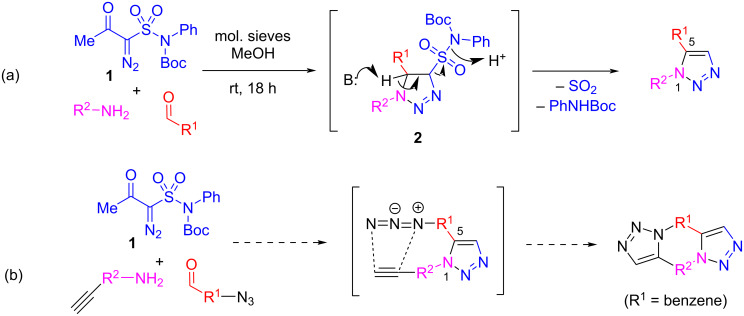
(a) Previously developed three-component approach to 1,5-disubstituted 1,2,3-triazoles; (b) double cycloaddition strategy investigated in this work.

Recently, we reported a novel, metal-free synthesis of 1,5-disubstituted 1,2,3-triazoles via a three-component reaction of α-acetyl-α-diazomethanesulfonamide (**1**) with aldehydes and amines [4]. The reaction proceeded, presumably, through the formation of the initial 1,2,3-triazoline adduct **2** [5] which underwent aromatization with the loss of sulfur dioxide and *N*-Boc-aniline. The multicomponent character and the fairly large scope of this reaction allows to place pairwise reactive groups in the aldehyde and the amine components, which would set a scene for further elaboration of the product’s molecular scaffold. Pondering various opportunities for post-condensational modifications of the 1,5-disubstituted 1,2,3-triazole core according to this strategy, we turned our attention to such powerful transformation as the azide–alkyne [3 + 2] cycloaddition (also known as the azide–alkyne click reaction) [6]. Indeed, if an alkyne and an azido group were strategically positioned within the structure of the amine and the aldehyde components for the reaction with **1**, subsequent intramolecular azide–alkyne cycloaddition would be a feasible event which would create a polycyclic bis-1,2,3-triazole framework (Figure 1). Herein, we report on a successful realization of this strategy.

## Results and Discussion

**Scheme 1:**
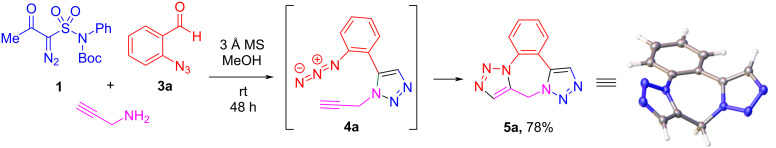
Results of a trial reaction between **1**, *o*-azidobenzaldehyde (**3a**) and propargylamine.

To test the possibility of a tandem double cycloaddition reaction between **1**, an alkyne-containing amine and an azide-containing aldehyde, we set up a reaction of **1** with *o*-azidobenzaldehyde (**3a**) and propargylamine. The reaction was allowed to go to completion in 48 h at room temperature whereupon the reaction mixture was absorbed on silica and subjected to column chromatography for isolation of the product. To our sheer amazement, the product turned out to be not the initial adduct **4a** but rather 9*H*-benzo[*f*]bis([1,2,3]triazolo)[1,5-*a*:1',5'-*d*][1,4]diazepine (**5a**), i.e., the product of the tandem three-component 1,2,3-triazole synthesis followed by intramolecular azide–alkyne click reaction which, apparently, proceeded at room temperature. Product **5a** was isolated in respectable 78% yield; therefore, the reaction conditions were not further optimized (Scheme 1). The structure of tetracyclic product **5a** was unequivocally confirmed by ^1^H and ^13^C NMR as well as single-crystal X-ray analysis.

**Figure 2:**
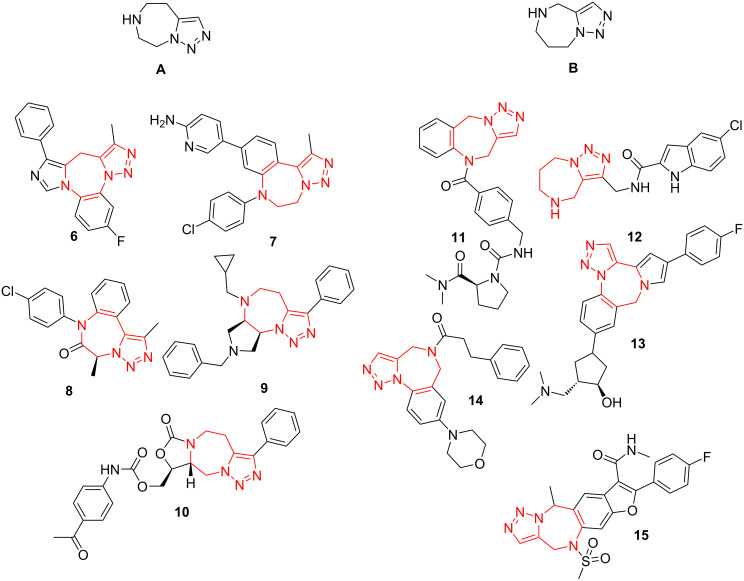
Diversely bioactive compounds based on scaffolds A and B.

Compound **5a** is representative of the hitherto undescribed bistriazole benzodiazepine scaffold. However, 5,6,7,8-tetrahydro-4*H*-[1,2,3]triazolo[1,5-*d*][1,4]diazepine (**A**) and 5,6,7,8-tetrahydro-4*H*-[1,2,3]triazolo[1,5-*a*][1,4]diazepine (**B**) scaffolds are of high medicinal importance, as evident from the literature. The range of biologically active compounds based on these two closely related scaffolds (both incorporated as fragments in the structure of compound **5a**) include compound **6** for the treatment of cognitive impairment [7], BET bromodomain inhibitors **7** [8] and **8** [9] for cancer treatment, σ_1_ receptor modulator **9** for diverse disorders [10] and bacterial regulatory RNA binder **10** [11] (scaffold A) as well as antidiuretic **11** [12], glycogen phosphorylase inhibitor **12** [13], MK2 kinase inhibitor **13** [14], ENL YEATS domain inhibitor **14** for leukemia treatment [15] and hepatitis C NS5B polymerase inhibitor **15** [16] (scaffold B, Figure 2).

**Scheme 2:**
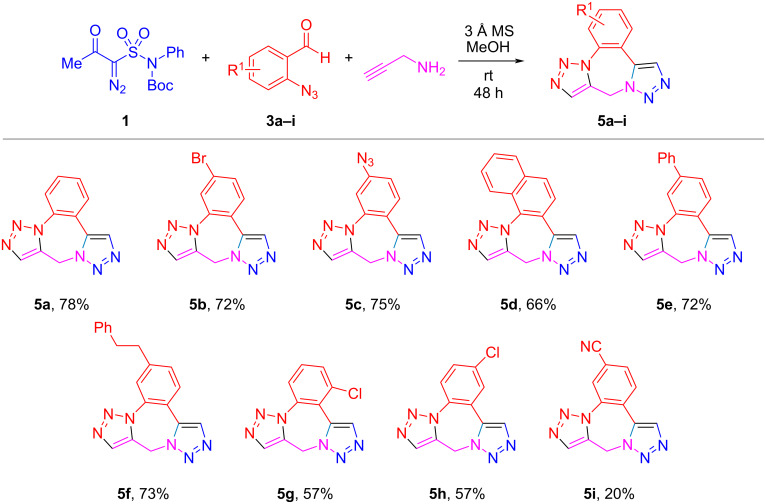
Three-component reaction of **1**, propargylamine and various *o*-azidobenzaldehydes.

The very fact that it was impossible to isolate intermediate **4a** from the reaction depicted in Scheme 1 speaks for the unusual facility with which the intramolecular azide–alkyne click reaction took place. Normally, intermolecular click reactions are copper-catalyzed [17–20]. Intramolecular positioning of the click reaction partners may eliminate the need for the metal-based catalyst but the reaction still requires thermal activation [21]. Thus, to our knowledge, the room temperature intramolecular azide–alkyne cycloaddition is unprecedented (plasmon-assisted click reaction at low temperature has been recently reported [22]). Excited by our initial finding, we tested various azide-containing aromatic aldehydes **3a–i** in the reaction with **1** and propargylamine (Scheme 2).

The yields of products **5** were generally fair to good for all the aldehydes tested, even for doubly azide-substituted aldehyde **3c**. However, the yield diminished somewhat for chloro-substituted aldehydes **5g–h**. Stronger electron-withdrawing cyano group (**3i**) lowered the product yields to 20% due to low conversion of **3i**.

**Figure 3:**
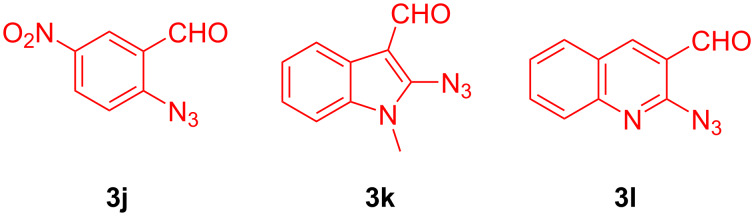
Aromatic azido aldehydes **3j–l** that failed to react with **1** and propargylamine.

Introducing even a stronger electron acceptor, a nitro group (aldehyde **3j**), led to only a trace amount of the respective product detected by ^1^H NMR analysis of the crude reaction mixture. Likewise, heterocyclic azido aldehydes **3k,l** failed to react with **1** and propargylamine (Figure 3).

**Scheme 3:**
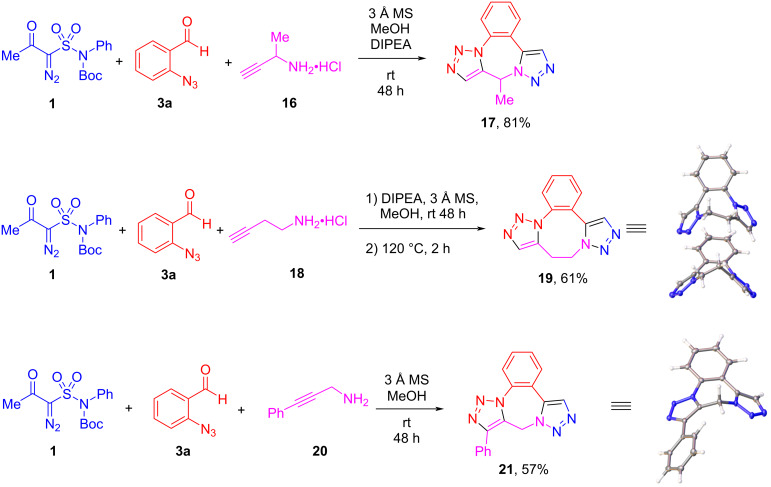
Variations of the amine component in the reactions with **1** and **3a**.

Having established the scope and limitations of the 9*H*-benzo[*f*]bis([1,2,3]triazolo)[1,5-*a*:1',5'-*d*][1,4]diazepine (**5**) synthesis, we proceeded to look at the variations in the alkyne-containing amine component for this transformation. Introduction of an α-methyl substitution in propargylamine (compound **16**) was tolerated and the respective reaction gave product **17** in a yield comparable to (and somewhat better than) that of unsubstituted compound **5a**. Homologation of propargylamine made a significant impact on the course of the reaction. The use of homopropargylamine (**18**) in the reaction of **1** with aldehyde **3a** abolished the facility of the intramolecular azide–alkyne click reaction which now required heating at 120 °C for 2 hours for the eight-membered (1,5-diazocane) ring to form (notably, the presence of the azide–alkyne intermediate before the click reaction was established by ^1^H NMR analysis of the reaction mixture). However, the product of this two-step, one-pot reaction (**19**) was isolated in respectable 61% yield. The structure of compound **19** was confirmed by the single-crystal X-ray analysis which demonstrated that the compound crystalized in two distinct conformers. Finally, we were curious to see if terminally substituted propargylamine **20** would react with **1** and aldehyde **3a** under the same reaction conditions. To our delight, the additional phenyl substituent did not dramatically influence the course of the reaction although the yield of product **21** was diminished compared to that of unsubstituted compound **5a**. The structure of compound **21** was also confirmed by the single-crystal X-ray analysis (Scheme 3).

All compounds were tested against lung cancer cell lines A549 and NCI-H460 and did not show any appreciable effect on cell proliferation in concentrations up to 250 μM. This validates these novel compounds as non-cytotoxic probes for interrogation of various biological targets.

## Conclusion

The previously described α-acetyl-α-diazomethanesulfonamide was employed in a three-component reaction with azide-containing benzaldehydes and propargylamines. Besides the initial formation of the triazole core, the reaction proceeded further, in uncatalyzed fashion at room temperature and yielded structurally intriguing bistriazoles whose structure was unequivocally confirmed by single-crystal X-ray analysis. Compounds are non-cytotoxic which makes them suitable for interrogation of various biological targets.

## Supporting Information

Deposition numbers 2183765 (for **5a**), 2183766 (for **19**) and 2183767 (for **21**) contain the supplementary crystallographic data for this paper. These data are provided free of charge by the joint Cambridge Crystallographic Data Centre and Fachinformationszentrum Karlsruhe Access Structures service http://www.ccdc.cam.ac.uk/structures.

File 1 General experimental information, X-ray crystallographic data, synthetic procedures, analytical data and NMR spectra for the reported compounds.
