# Supplementary material for: A novel bis-triazole scaffold accessed via two tandem [3 + 2] cycloaddition events including an uncatalyzed, room temperature azide–alkyne click reaction
Source: Beilstein J Org Chem. 2022 Dec 2;18:1636–41. doi: 10.3762/bjoc.18.175 (PMC9727270; doi:10.3762/bjoc.18.175)
Supplement: File 1 — General experimental information, X-ray crystallographic data, synthetic procedures, analytical data and NMR spectra for the reported compounds. [file Beilstein_J_Org_Chem-18-1636-s001.pdf]

BEILSTEIN JOURNAL OF ORGANIC CHEMISTRY

Supporting Information

for

A novel bis-triazole scaffold accessed via two tandem [3 + 2] cycloaddition events including an uncatalyzed, room temperature azide–alkyne click reaction

Ksenia Malkova, Andrey Bubyrev, Vasilisa Krivovicheva, Dmitry Dar'in, Alexander Bunev and Mikhail Krasavin

Beilstein J. Org. Chem. **2022,** *18,* 1636–1641. doi:10.3762/bjoc.18.175

General experimental information, X-ray crystallographic data, synthetic procedures, analytical data and NMR spectra for the reported compounds

License and Terms: This is a supporting information file under the terms of the Creative Commons Attribution License (https://creativecommons.org/licenses/by/4.0). Please note that the reuse, redistribution and reproduction in particular requires that the author(s) and source are credited and that individual graphics may be subject to special legal provisions.
The license is subject to the *Beilstein Journal of Organic Chemistry* terms and conditions: (https://www.beilstein-journals.org/bjoc/terms)

Table of contents

I. Materials and methods S2

II. Synthesis of the starting materials S3

III. General procedure (GP) for the synthesis of 5a–i and 17,19,21 .... S10

IV. Biological assay S13

V. NMR spectra S14

VI. X-ray crystallographic data S26

VII. References S30

I. Materials and methods

All commercial reagents were used without purification. NMR spectra were recorded using a Bruker Avance III spectrometer in $CDCl_3$ or DMSO-d_6 (^{1}H: 400.13 MHz, ^{13}C: 100.61 MHz and 125.73 MHz). All chemical shifts are reported in parts per million (ppm). The residual solvent peak was used as internal standard: $CDCl_3$(7.26 for ^{1}H and 77.16 ppm for ^{13}C), DMSO-d_6 (2.50 for ^{1}H and 39.52 ppm for ^{13}C). Standard abbreviations were used in the description of resonances. Coupling constants (*J*) are quoted to the nearest 0.1 Hz. Mass spectra were recorded with a HRMS-ESI-qTOF spectrometer (electrospray ionization mode, positive ion detection). Melting points were determined with a melting point apparatus Stuart SMP 10 in open capillary tubes. Single crystal X-ray data were obtained using an Agilent Technologies SuperNova Atlas and an Agilent Technologies Xcalibur Eos diffractometer at a temperature of 100 K. Analytical thin layer chromatography was carried out on UV-254 silica gel plates using appropriate eluents. Compounds were visualized with short wave length UV light. Column chromatography was performed using silica gel Merk grade 60 (0.040−0.063 mm) 230−400 mesh.

II. Synthesis of the starting materials

Synthesis of *1*

Tert-butyl (1-diazo-2-oxopropyl)sulfonyl(phenyl)carbamate **1** was synthesized as described previously.[1]

Synthesis of *o*-azidobenzaldehydes 3a–l

*2-Azidobenzaldehyde (**3a**)*

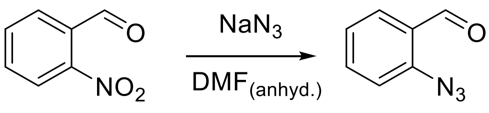

S1 **3a**

2-Azidobenzaldehyde (**3a**) was prepared from 2-nitrobenzaldehyde (**S1**) according to a literature technique.[2] Yield of **3a**: 1.05 g (43%). The spectroscopic data matched those reported in the literature.[2]

*2-Azido-4-bromobenzaldehyde (**3b**)*

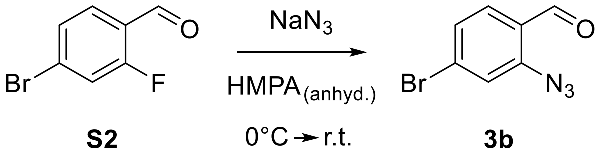

S2 **3b**

To a solution of 4-bromo-2-fluorobenzaldehyde (**S2**, 1g, 4.9 mmol, 1 equiv) in 8 mL of anhydrous HMPA was added sodium azide (320 mg, 4.9 mmol, 1 equiv) at 0 °C (ice/water bath). The reaction was stirred at room temperature for 72 h. The mixture was poured into ice cold water. The resulting precipitate was filtered, washed with ice cold water and dried over P_2O_5 in vacuum desiccator. The crude product was purified by column chromatography on silica gel (gradient elution: hexane/ethyl acetate 100:0 → 97:3). Yield of **3b**: 865 mg (78%). The spectroscopic data matched those reported in the literature.[3]

*2,4-Diazidobenzaldehyde (**3c**)*

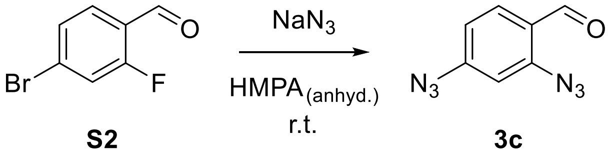

S2 **3c**

To a solution of 4-bromo-2-fluorobenzaldehyde (**S2**, 676 mg, 3.3 mmol, 1 equiv) in 8 mL of anhydrous HMPA was added sodium azide (649 mg, 9.9 mmol, 3 equiv). The reaction was stirred at room temperature overnight. The mixture was poured into ice cold water. The resulting precipitate was filtered, washed with ice cold water and dried over P_2O_5 in vacuum desiccator. The precipitate obtained was recrystallized from isopropyl alcohol. Yield of **3c**: 503 mg (80%). The spectroscopic data matched those reported in the literature.[4]

*1-Azido-2-naphthaldehyde (**3d**)*

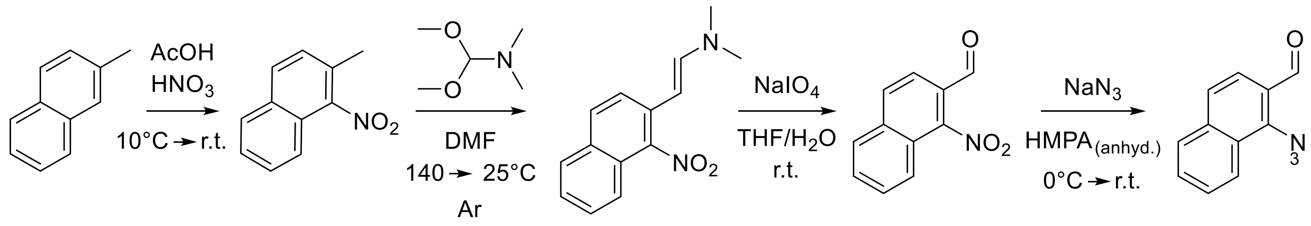

S3 **S4** **S5** **S6** **3d**

2-Methyl-1-nitronaphthalene (**S4**) was prepared from 2-methylnaphthalene (**S3**) according to a literature technique.[5] Yield of **S4**: 2.99 g (46%).

The syntheses of (*E*)-*N*,*N*-dimethyl-2-(1-nitronaphthalen-2-yl)ethen-1-amine (**S5**) and 1-nitro-2-naphthaldehyde (**S6**) were based on a literature procedure.[6] Yield of **S5**: 3.04 g (78%). Yield of **S6**: 1.66 g (99%). The spectroscopic data matched those reported in the literature.[6]

1-Azido-2-naphthaldehyde (**3d**) was prepared in accordance with a literature technique.[7] Yield of **3d**: 235 mg (60%). The spectroscopic data matched those reported in the literature.[7]

*3-Azido-[1,1'-biphenyl]-4-carbaldehyde (**3e**)*

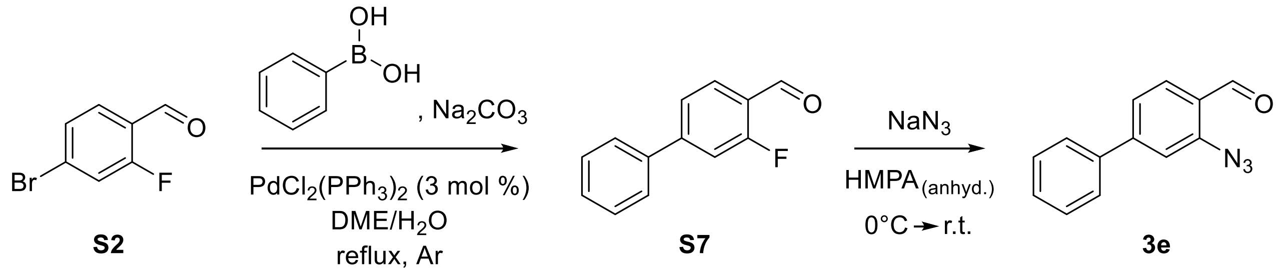

S2 **S7** **3e**

3-Fluoro-[1,1'-biphenyl]-4-carbaldehyde (**S7**) was prepared from 4-bromo-2-fluorobenzaldehyde (**S2**) according to a literature technique.[8] Yield of **S7**: 496 mg (72%). The spectroscopic data matched those reported in the literature.[9]

To a solution of **S7** (480 mg, 2.4 mmol, 1 equiv) in 6 mL of anhydrous HMPA was added sodium azide (312 mg, 4.8 mmol, 2 equiv) at 0 °C (ice/water bath). The reaction was stirred at room temperature for 72 h. Another portion of sodium azide (156 mg, 2.4 mmol, 1 equiv) was added and the reaction mixture was stirred at 40 °C overnight. The mixture was poured into ice cold water. The resulting precipitate was filtered, washed with ice cold water and dried over P_2O_5 in vacuum desiccator. The precipitate was triturated with hexane twice and dried in vacuo.

Yield of **3e**: 426 mg (76%). Beige powder. ^{1}H NMR (400 MHz, $CDCl_3$) δ 10.37 (s, 1H), 7.95 (d, *J* = 8.0 Hz, 1H), 7.69 – 7.56 (m, 2H), 7.56 – 7.39 (m, 5H). ^{13}C{^{1}H} NMR (101 MHz, $CDCl_3$) δ 188.3, 148.7, 143.4, 139.1, 129.7, 129.3, 129.1, 127.4, 125.9, 124.0, 117.5. HRMS (ESI) m/z: $[M+H]^+$ Calcd for $C_{13}H_{10}N_3O$ $[M+H]^+$ 224.0818; Found 224.0820.

*2-Azido-4-phenethylbenzaldehyde (**3f**)*

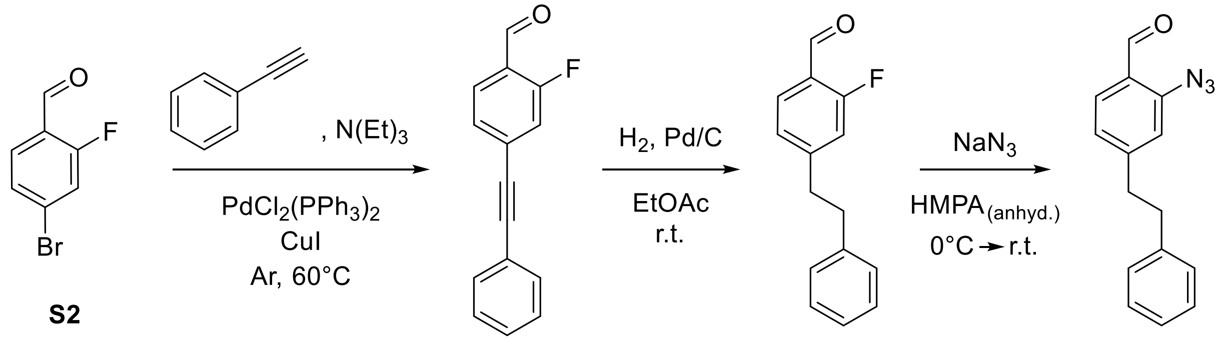

S2 **S8** **S9** **3f**

The synthesis of **S8** was based on a literature method.[10] Argon was bubbled through a solution of **S2** (1 g, 4.9 mmol, 1 equiv) in 43 mL of $N(Et)_3$. To the solution were added $PdCl_2(PPh_3)_2$ (70 mg, 0.10 mmol, 2 mol %), CuI (38 mg, 0.20 mmol, 4 mol %), and phenylacetylene (705 µL, 6.4 mmol, 1.3 equiv). The mixture was stirred at 60 °C under an inert atmosphere overnight. The solvent was removed in vacuo. The residue was chromatographed on silica gel (gradient elution: hexane/ethyl acetate 100:0 → 95:5). The product was recrystallized from cyclohexane. Yield of **S8**: 615 mg (56%). Beige powder. ^{1}H NMR (400 MHz, $CDCl_3$) δ 10.34 (s, 1H), 7.85 (t, *J* = 7.6 Hz, 1H), 7.61 – 7.49 (m, 2H), 7.45 – 7.34 (m, 4H), 7.31 (dd, *J* = 10.9, 1.4 Hz, 1H). ^{13}C{^{1}H} NMR (101 MHz, $CDCl_3$) δ 186.5 (d, C–F, *J* = 6.3 Hz), 164.4 (d, C–F, *J* = 258.8 Hz), 132.0, 131.7 (d, C–F, *J* = 10.7 Hz), 129.4, 128.7 (d, C–F, *J* = 2.8 Hz), 128.7, 128.0 (d, C–F, *J* = 3.4 Hz), 123.6 (d, C–F, *J* = 8.4 Hz), 122.2, 119.3 (d, C–F, *J* = 22.3 Hz), 94.7, 87.6 (d, C–F, *J* = 3.1 Hz). ^{19}F{^{1}H} NMR (376 MHz, $CDCl_3$) δ -122.2. HRMS (ESI) m/z: $[M+H]^+$ Calcd for $C_{15}H_{10}FO$ $[M+H]^+$ 225.0710; Found 225.0712.

Argon was bubbled through a solution of **S8** (575 mg, 2.6 mmol, 1 equiv) in 10 mL of EtOAc. To the solution was added Pd/C (40 mg). H_2 gas was bubbled through the mixture. The mixture was stirred at room temperature under a hydrogen atmosphere overnight. The reaction solution was separated from the catalyst by filtration through a membrane (0.45 µm pore size). The volatiles were removed in vacuo to afford **S9**. Yield of **S9**: 561 mg (96%). The product was used without further purification.

To a solution of **S9** (561 g, 2.5 mmol, 1 equiv) in 5 mL of anhydrous HMPA was added sodium azide (320 mg, 4.9 mmol, 2 equiv) at 0 °C (ice/water bath). The reaction was stirred at room temperature for 72 h. Another portion of sodium azide (80 mg, 1.2 mmol, 0.5 equiv) was added to the mixture. The reaction was stirred at 50 °C overnight. To the reaction mixture was added water. The product was extracted EtOAc (2 × 20 mL). The combined organic layers were washed with water (2×20 mL) and brine (1 × 30 mL). The extract was dried over anhyd. Na_2SO_4 and concentrated in vacuo. The product was purified by column chromatography on silica gel (gradient elution: hexane/ethyl acetate 100:0 → 95:5). Yield of **3f**: 282 mg (46%). Beige powder. ^{1}H NMR (400 MHz, $CDCl_3$) δ 10.29 (s, 1H), 7.79 (d, *J* = 8.0 Hz, 1H), 7.33 – 7.27 (m, 2H), 7.25 – 7.19 (m, 1H), 7.15 (dd, *J* = 7.0, 1.8 Hz, 2H), 7.03 (dd, *J* = 8.0, 1.5 Hz, 1H), 6.95 (d, *J* = 1.5 Hz, 1H), 3.06 – 2.91 (m, 4H). ^{13}C{^{1}H} NMR (101 MHz, $CDCl_3$) δ 188.4, 150.5, 142.9, 140.6, 129.3, 128.6, 128.6,

126.5, 125.6, 125.4, 119.1, 38.1, 37.2. HRMS (ESI) m/z: $[M+H]^+$ Calcd for $C_{15}H_{14}N_3O$ $[M+H]^+$ 252.1131; Found 252.1131.

*2-Azido-6-chlorobenzaldehyde (**3g**)*

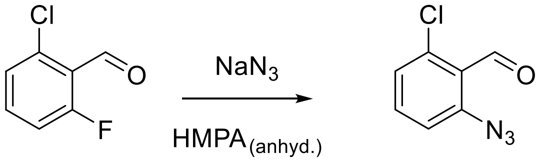

S10 **3g**

To a solution of 2-chloro-6-fluorobenzaldehyde (**S10**, 700 mg, 4.4 mmol, 1 equiv) in 5 mL of anhydrous HMPA was added sodium azide (287 mg, 4.4 mmol, 1 equiv) at 0 °C (ice/water bath). The reaction was stirred at room temperature for 72 h. The mixture was poured into ice cold water. The resulting precipitate was filtered, washed with ice cold water and dried over P_2O_5 in vacuum desiccator. The crude product was purified by column chromatography on silica gel (gradient elution: hexane/ethyl acetate 100:0 → 96:4). Yield of **3g**: 667 mg (83%). The spectroscopic data matched those reported in the literature.[7]

*2-Azido-5-chlorobenzaldehyde (**3h**)*

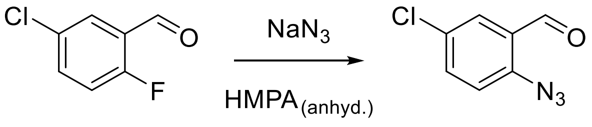

S11 **3h**

To a solution of 5-chloro-2-fluorobenzaldehyde (**S11**, 700 mg, 4.4 mmol, 1 equiv) in 5 mL of anhydrous HMPA was added sodium azide (287 mg, 4.4 mmol, 1 equiv) at 0 °C (ice/water bath). The reaction was stirred at room temperature for 72 h. The mixture was poured into ice cold water. The resulting precipitate was filtered, washed with ice cold water and dried over P_2O_5 in vacuum desiccator. Yield of **3h**: 694 mg (87%). The spectroscopic data matched those reported in the literature.[11]

*3-Azido-4-formylbenzonitrile (**3i**)*

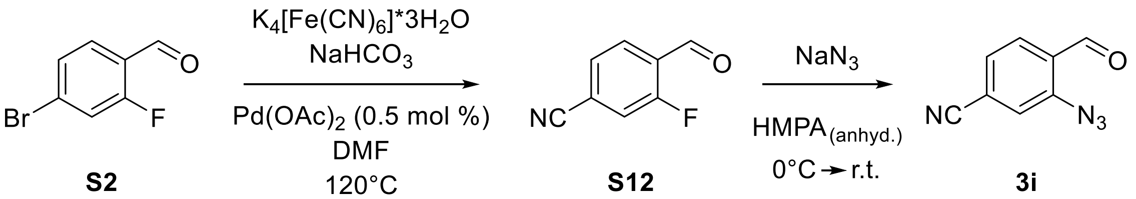

S2 **S12** **3i**

3-Fluoro-4-formylbenzonitrile (**S12**) was prepared from **S2** according to a literature technique.[12] The product was purified by recrystallization from cyclohexane/pentane/CH_2Cl_2 system. Yield of **S12**: 218 mg (42%). The spectroscopic data matched those reported in the literature.[13]

To a solution of 4-bromo-2-fluorobenzaldehyde (**S12**, 216 mg, 1.45 mmol, 1 equiv) in 4 mL of anhydrous HMPA was added sodium azide (188 mg, 2.9 mmol, 1 equiv) at 0 °C (ice/water bath). The reaction was stirred at room temperature for 72 h. The mixture was poured into ice cold water. The resulting precipitate was filtered, washed with ice cold water and dried over P_2O_5 in vacuum desiccator. The crude product was purified by column chromatography on silica gel (gradient elution: hexane/ethyl acetate 100:0 → 90:10). Yield of **3i**: 75 mg (44%). Light orange powder. ^{1}H NMR (400 MHz, $CDCl_3$) δ 10.38 (s, 1H), 7.98 (d, J = 8.0 Hz, 1H), 7.56 (d, J = 1.4 Hz, 1H), 7.51 (dd, J = 8.0, 1.4 Hz, 1H). ^{13}C{^{1}H} NMR (101 MHz, $CDCl_3$) δ187.3, 143.8, 129.9, 129.4, 128.2, 122.8, 118.7, 117.0. HRMS (ESI) m/z: $[M-N_2+H]^+$ Calcd for $C_8H_5N_2O$ $[M-N_2+H]^+$145.0396; Found 145.0399.

*2-Azido-5-nitrobenzaldehyde (**3j**)*

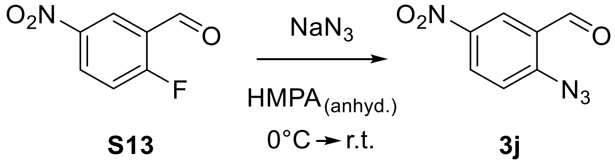

S13 **3j**

To a solution of 2-fluoro-5-nitrobenzaldehyde (**S13**, 700 mg, 4.1 mmol, 1 equiv) in 5 mL of anhydrous HMPA was added sodium azide (283 mg, 4.35 mmol, 1.05 equiv) at 0 °C (ice/water bath). The reaction was stirred at 0 °C for 6 h. The mixture was poured into ice cold water. The resulting precipitate was filtered, washed with ice cold water and dried over P_2O_5 in vacuum desiccator. Yield of **3j**: 694 mg (87%). The spectroscopic data matched those reported in the literature.[14]

*2-Azido-1-methyl-1H-indole-3-carbaldehyde (**3k**)*

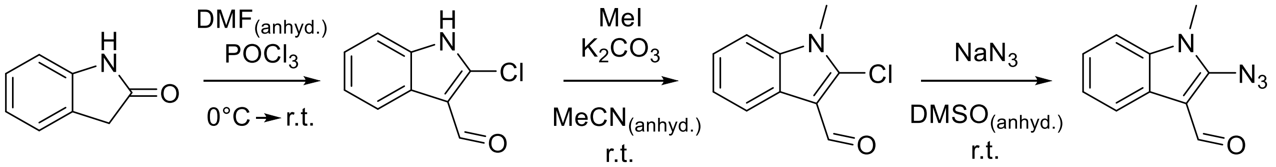

S14 **S15** **S16** **3k**

2-Chloro-1*H*-indole-3-carbaldehyde (**S15**) and 2-chloro-1-methyl-1*H*-indole-3-carbaldehyde (**S16**) were synthesized according to a literature technique.[15] Yield of **S15**: 1.51 g (74%). Yield of **S16**: 430 mg (40%). The spectroscopic data matched those reported in the literature.[15]

2-Azido-1-methyl-1*H*-indole-3-carbaldehyde (**3k**) was prepared from **S16** in accordance with a literature procedure.[16] Yield of **3k**: 358 mg (81%). The product was used without further purification (7mol % of **S16** according to NMR). The spectroscopic data matched those reported in the literature.[16]

*2-Azidoquinoline-3-carbaldehyde (**3l**)*

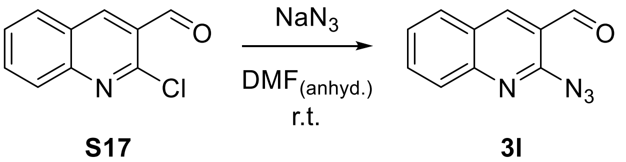

2-Azidoquinoline-3-carbaldehyde (**3l**) was prepared from 2-chloroquinoline-3-carbaldehyde **S17** according to a literature technique.[17] Yield of **3l**: 825 mg (80%). The spectroscopic data matched those reported in the literature.[17]

The synthesis of propargyl amines 16, 18, 20

*But-3-yn-2-amine hydrochloride (**16**)*

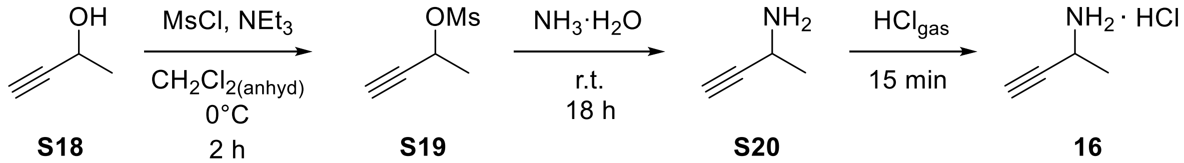

But-3-yn-2-yl methanesulfonate (**S19**), but-3-yn-2-amine (**S20**) and but-3-yn-2-amine hydrochloride **16** were prepared according to a literature technique.[18] Yield of **16**: 1.09 g (30 %). The spectroscopic data matched those reported in the literature.[19]

*But-3-yn-1-amine hydrochloride (**18**)*

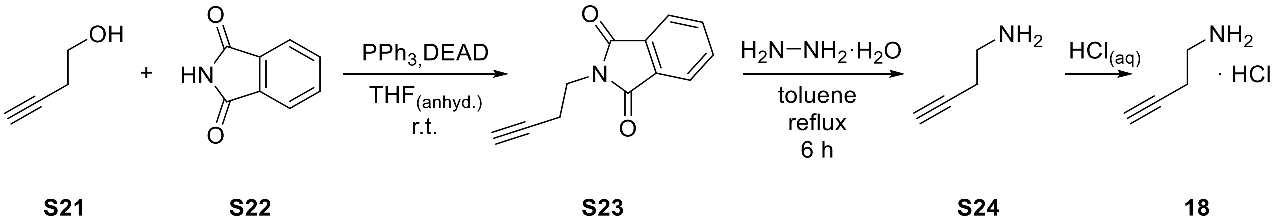

Phthalimide (**S22**, 2.94 g, 20 mmol, 1 equiv) and PPh_3(5.24 g, 20 mmol, 1 equiv) were dissolved in 60 mL of dry THF. To the solution were added but-3-yn-1-ol (**S21**, 1.52 mL, 20 mmol, 1 equiv) and DEAD (3.15 mL, 20 mmol, 1 equiv). The reaction mixture was stirred at room temperature overnight. The solvent was removed in vacuo. The residue was chromatographed on silica gel (gradient elution: hexane/chloroform 95:5 → 50:50) to afford **S23**. Yield: 3.35g. The product was used without further purification (8.5 mol % of $OPPh_3$, NMR data).

But-3-yn-1-amine hydrochloride **18** was prepared according to a literature technique. Yield of **18**: 305 mg (60%). The spectroscopic data matched those reported in the literature.[21]

*3-Phenylprop-2-yn-1-amine (**20**)*

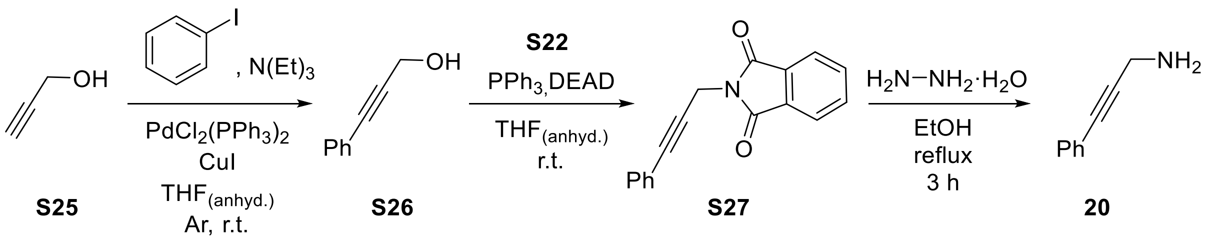

Argon was bubbled through a solution of phenyl iodide (1.37mL, 12.3 mmol, 1 equiv) in 25 mL of THF. To the solution were added $PdCl_2(PPh_3)_2$ (129 mg, 0.18 mmol, 1.5 mol %), CuI (35 mg, 0.18 mmol, 1.5 mol %), and $N(Et)_3$ (6.83mL, 49.0 mmol, 4 equiv). The mixture was stirred for 5 min. Propargylic alcohol **S25** (785 μL, 13.5 mmol, 1.1 equiv) was added to the reaction system. The mixture was stirred at room temperature under an inert atmosphere overnight. The reaction mixture was filtered through a plug of Celite. The solvent was removed in vacuo. The residue was dissolved in EtOAc and washed with 1 M HCl twice, water and brine. The extract was dried over anhyd. Na_2SO_4 and concentrated in vacuo to give S26 in quantitative yield. The crude product was used without further purification.

To a solution of **S26** in 30 mL of anhydrous THF were added phthalimide (**S22**, 1.91 g, 13 mmol, 1.1 equiv), PPh_3 (5.08 g, 19.4 mmol, 1.6 equiv), and DEAD (3.05 mL, 19.4 mmol, 1.6 equiv). The solution was stirred overnight. The solvent was removed in vacuo. The residue was chromatographed on silica gel (gradient elution: hexane/CH_2Cl_2 100:0 → 50:50). Yield of **S27**: 2.42 g (73%). The product was used without further purification. The spectroscopic data matched those reported in the literature.[22]

3-Phenylprop-2-yn-1-amine (**20**) was prepared from **S27** according to a literature technique.[22] Yield of **20**: 260 mg (57%). The product was used without further purification (7.5 mol % of diethyl hydrazine-1,2-dicarboxylate, NMR data). The spectroscopic data matched those reported in the literature.[22]

III. General procedure (GP) for the synthesis of 5a–i and 17,19,21

Tert-butyl (1-diazo-2-oxopropyl)sulfonyl(phenyl)carbamate **1** (300 mg, 0.88 mmol, 1.5 equiv), azidoaldehyde **3a** (0.59 mmol, 1.0 equiv), molecular sieves (200 mg), and methanol (8 ml) were placed in screw-capped glass vial. The reaction mixture was stirred for 5 min. Propargyl amine (57 μL, 0.88 mmol, 1.5 equiv) was added to the mixture. The resulting suspension was stirred for 48 h at room temperature. The mixture was adsorbed onto silica and chromatographed with a hexanes/chloroform/ethyl acetate system using gradient elution (equilibration - 50:50:0, gradient - 45:45:10 → 20:20:60). The solid obtained was triturated with diethyl ether (2 mL) twice. The residue was dried in vacuo to afford benzobistriazolodiazepine **5a–i.**

*9H-Benzo[f]bis([1,2,3]triazolo)[1,5-a:1',5'-d][1,4]diazepine (**5a**)*

Yield: 58 mg (78%) from 0.33 mmol of **3a**. Beige powder; mp 245-246 °C. ^{1}H NMR (400 MHz, DMSO-d_6) δ 8.28 (s, 1H), 8.16 (dd, J = 8.1, 1.4 Hz, 1H), 8.06 (s, 1H), 7.95 (dd, J = 7.7, 1.7 Hz, 1H), 7.77 (ddd, J = 8.1, 7.7, 1.7 Hz, 1H), 7.71 (td, J = 7.7, 1.4 Hz, 1H), 6.05 (s, 2H).^{13}C{^{1}H} NMR (101 MHz, DMSO-d_6) δ 134.2, 133.7, 133.3, 132.6, 132.6, 131.2, 130.0, 129.7, 123.9, 117.8, 40.7. HRMS (ESI) m/z: [M+H]$^+$ Calcd for $C_{11}H_9N_6$ [M+H]$^+$ 225.0883; Found 225.0883.

*3-Bromo-9H-benzo[f]bis([1,2,3]triazolo)[1,5-a:1',5'-d][1,4]diazepine (**5b**)*

Yield: 128 mg (72%). White powder; mp >299 °C. ^{1}H NMR (400 MHz, DMSO-d_6) δ 8.33 (d, J = 1.9 Hz, 1H), 8.31 (s, 1H), 8.06 (s, 1H), 7.95 (dd, J = 8.5, 1.9 Hz, 1H), 7.91 (d, J = 8.5 Hz, 1H), 6.05 (s, 2H). ^{13}C{^{1}H} NMR (101 MHz, DMSO-d_6) δ 133.9, 133.5, 133.45, 133.44, 132.8, 132.5, 131.7, 126.3, 123.6, 117.1, 40.7. HRMS (ESI) m/z: [M+H]$^+$ Calcd for $C_{11}H_8BrN_6$ [M+H]$^+$ 302.9988; Found 302.9985.

*3-Azido-9H-benzo[f]bis([1,2,3]triazolo)[1,5-a:1',5'-d][1,4]diazepine (**5c**)*

Yield: 117 mg (75%). Light orange powder; decomp. 205-210 °C. ^{1}H NMR (400 MHz, DMSO-d_6) δ 8.27 (s, 1H), 8.07 (s, 1H), 7.97 (d, J = 8.5 Hz, 1H), 7.81 (d, J = 2.4 Hz, 1H), 7.50 (dd, J = 8.5, 2.4 Hz, 1H), 6.04 (s, 2H). ^{13}C{^{1}H} NMR (101 MHz, DMSO-d_6) δ 142.4, 133.7, 133.6, 133.5, 133.3, 132.8, 131.7, 120.4, 114.3, 114.1, 40.6. HRMS (ESI) m/z: [M+H]$^+$ Calcd for $C_{11}H_8N_9$ [M+H]$^+$ 266.0897; Found 266.0896.

*9H-Naphtho[2,1-f]bis([1,2,3]triazolo)[1,5-a:1',5'-d][1,4]diazepine (**5d**)*

Yield: 106 mg (66%). Beige powder; mp 266-267 °C. ^{1}H NMR (400 MHz, DMSO-d_6) δ 8.39 (s, 1H), 8.30 (d, J = 8.6 Hz, 1H), 8.25 (dd, J = 7.7, 1.8 Hz, 1H), 8.20 – 8.16 (m, 1H), 8.15 (s, 1H), 7.95 (d, J = 8.6 Hz, 1H), 7.80 – 7.69 (m, 2H), 6.44 (d, J = 15.2 Hz, 1H), 5.70 (d, J = 15.2 Hz, 1H). ^{13}C{^{1}H} NMR (101 MHz, DMSO-d_6) δ 136.1, 134.3, 134.3, 133.9, 131.6, 130.4, 128.8, 128.3, 128.16, 128.15, 127.2, 126.2, 125.2, 117.6, 40.7. HRMS (ESI) m/z: [M+H]$^+$ Calcd for $C_{15}H_{11}N_6$ [M+H]$^+$ 275.1040; Found 275.1042.

*3-Phenyl-9H-benzo[f]bis([1,2,3]triazolo)[1,5-a:1',5'-d][1,4]diazepine (**5e**)*

Yield: 127 mg (72%). White powder; mp 237-238 °C. ^{1}H NMR (400 MHz, DMSO-d_6) δ 8.40 (s, 1H), 8.33 (s, 1H), 8.09 (s, 1H), 8.04 (s, 2H), 7.92 – 7.82 (m, 2H), 7.56 (dd, J = 7.7, 7.3 Hz, 2H), 7.49 (t, J = 7.3 Hz, 1H), 6.08 (s, 2H). ^{13}C{^{1}H} NMR (101 MHz, DMSO-d_6) δ 142.8, 137.8, 134.0, 133.7, 133.3, 133.1, 132.7, 130.7, 129.3, 128.7, 127.7, 126.9, 121.6, 116.7, 40.7. HRMS (ESI) m/z: $[M+H]^+$Calcd for $C_{17}H_{13}N_6$ $[M+H]^+$ 301.1196; Found 301.1197.

*3-Phenethyl-9H-benzo[f]bis([1,2,3]triazolo)[1,5-a:1',5'-d][1,4]diazepine (**5f**)*

Yield: 153 mg (73%). White powder; mp 183-184 °C. ^{1}H NMR (400 MHz, DMSO-d_6) δ 8.24 (s, 1H), 8.09 (d, J = 1.7 Hz, 1H), 8.05 (s, 1H), 7.85 (d, J = 8.0 Hz, 1H), 7.59 (dd, J = 8.0, 1.7 Hz, 1H), 7.34 – 7.25 (m, 4H), 7.24 – 7.15 (m, 1H), 6.02 (s, 2H), 3.14 – 3.05 (m, 2H), 3.04 – 2.95 (m, 2H). ^{13}C{^{1}H} NMR (101 MHz, DMSO-d_6) δ 145.5, 141.0, 134.2, 133.4, 133.2, 132.6, 132.5, 129.9, 129.8, 128.4, 128.3, 126.0, 123.6, 115.5, 40.6, 36.44, 36.43. HRMS (ESI) m/z: $[M+H]^+$ Calcd for $C_{19}H_{17}N_6$ $[M+H]^+$ 329.1509; Found 329.1510.

*1-Chloro-9H-benzo[f]bis([1,2,3]triazolo)[1,5-a:1',5'-d][1,4]diazepine (**5g**)*

Yield: 86 mg (57%). White powder; mp 245-246 °C. ^{1}H NMR (400 MHz, DMSO-d_6) δ 8.33 (s, 1H), 8.11 – 8.01 (m, 2H), 7.93 (dd, J = 8.1, 1.3 Hz, 1H), 7.78 (t, J = 8.1 Hz, 1H), 6.42 (s, 1H), 5.73 (s, 1H). ^{13}C{^{1}H} NMR (101 MHz, DMSO-d_6) δ 136.6, 134.9, 134.8, 133.0, 132.2, 132.0, 131.1, 129.5, 123.9, 117.5, 40.7. HRMS (ESI) m/z: $[M+H]^+$ Calcd for $C_{11}H_8ClN_6$ $[M+H]^+$ 259.0493; Found 259.0496.

*2-Chloro-9H-benzo[f]bis([1,2,3]triazolo)[1,5-a:1',5'-d][1,4]diazepine (**5h**)*

Yield: 86 mg (57%). White powder; mp 294-295 °C. ^{1}H NMR (400 MHz, DMSO-d_6) δ 8.37 (s, 1H), 8.17 (d, J = 8.8 Hz, 1H), 8.09 (d, J = 2.4 Hz, 1H), 8.06 (s, 1H), 7.83 (dd, J = 8.8, 2.4 Hz, 1H), 6.06 (s, 2H). ^{13}C{^{1}H} NMR (101 MHz, DMSO-d_6) δ 134.3, 134.0, 133.3, 133.1, 132.7, 131.4, 130.9, 129.3, 125.8, 119.6, 40.7. HRMS (ESI) m/z: $[M+H]^+$ Calcd for $C_{11}H_8ClN_6$ $[M+H]^+$ 259.0493; Found 259.0494.

*9H-benzo[f]bis([1,2,3]triazolo)[1,5-a:1',5'-d][1,4]diazepine-3-carbonitrile (**5i**)*

Yield: 15 mg (20%) from 0.30 mmol of **3i**. White powder; mp >299 °C. ^{1}H NMR (400 MHz, DMSO-d_6) δ 8.65 (d, J = 1.6 Hz, 1H), 8.41 (s, 1H), 8.20 (dd, J = 8.2, 1.6 Hz, 1H), 8.16 (d, J = 8.2 Hz, 1H), 8.09 (s, 1H), 6.11 (s, 2H). ^{13}C{^{1}H} NMR (126 MHz, DMSO-d_6) δ 135.0, 133.5, 133.2, 133.0, 132.8, 131.2, 127.8, 122.2, 117.3, 113.4, 40.9. HRMS (ESI) m/z: $[M+H]^+$ Calcd for $C_{12}H_8N_7$ $[M+H]^+$ 250.0836; Found 250.0831.

*9-Methyl-9H-benzo[f]bis([1,2,3]triazolo)[1,5-a:1',5'-d][1,4]diazepine (**17**)*

But-3-yn-2-amine hydrochloride **16** (93 mg, 0.88 mmol, 1.5 equiv) was dissolved in methanol (8 mL) and DIPEA (154 μL, 0.88 mmol, 1.5 equiv) was added. The resulting solution was stirred for 20 min at room temperature. *tert*-Butyl (1-diazo-2-oxopropyl)sulfonyl(phenyl)carbamate (**1**, 300 mg, 0.88 mmol, 1.5 equiv), *o*-azidobenzaldehyde **3a** (87 mg, 0.59 mmol, 1.0 equiv), and molecular sieves 3 Å (200 mg) were added. The resulting suspension was stirred for 48 h at room temperature. The mixture was adsorbed onto silica and chromatographed with a hexanes/chloroform/ethyl

acetate system using gradient elution (equilibration - 50:50:0, gradient - 45:45:10 → 20:20:60). The solid obtained was triturated with diethyl ether (2 mL) twice. The residue was dried in vacuo to afford **17.**

Yield: 114 mg (81%). White powder; mp 194-196 °C. ^{1}H NMR (400 MHz, DMSO-d_6) δ 8.30 (s, 1H), 8.17 (dd, J = 8.0, 1.4 Hz, 1H), 8.07 (s, 1H), 7.96 (dd, J = 7.7, 1.6 Hz, 1H), 7.80 (ddd, J = 8.0, 7.7, 1.6 Hz, 1H), 7.73 (td, J = 7.7, 1.4 Hz, 1H), 6.32 (q, J = 7.0 Hz, 1H), 1.89 (d, J = 7.0 Hz, 3H). ^{13}C{^{1}H} NMR (101 MHz, DMSO-d_6) δ 137.4, 134.0, 133.5, 132.4, 131.6, 131.3, 129.8, 129.7, 123.7, 117.9, 48.6, 16.2. HRMS (ESI) m/z: $[M+H]^+$ Calcd for $C_{12}H_{11}N_6$ $[M+H]^+$ 239.1040; Found 239.1045.

*9,10-Dihydrobenzo[c]bis([1,2,3]triazolo)[1,5-a:1',5'-e][1,5]diazocine (**19**)*

But-3-yn-1-amine hydrochloride **18** (93 mg, 0.88 mmol, 1.5 equiv) was dissolved in methanol (8 mL) and DIPEA (154 µL, 0.88 mmol, 1.5 equiv) was added. The resulting solution was stirred for 20 min at room temperature. *tert*-Butyl(1-diazo-2-oxopropyl)sulfonyl(phenyl)carbamate **1** (300 mg, 0.88 mmol, 1.5 equiv), *o*-azidobenzaldehyde (**3a**, 87 mg, 0.59 mmol, 1.0 equiv), and molecular sieves 3 Å (200 mg) were added. The resulting suspension was stirred for 48 h at room temperature andthen 2 h at 120 °C. The mixture was adsorbed onto silica and chromatographed with a hexanes/chloroform/ethyl acetate system using gradient elution (equilibration - 50:50:0, gradient - 45:45:10 → 20:20:60). The solid obtained was triturated with diethyl ether (2 mL) twice. The residue was dried in vacuo to afford **19.**

Yield: 85 mg (61%). White powder; mp 168-169 °C. ^{1}H NMR (400 MHz, DMSO-d_6) δ 7.88 – 7.71 (m, 5H), 7.68 (dd, J = 7.4, 1.8 Hz, 1H), 4.97 (s, 1H), 4.52 (s, 1H), 3.66 (s, 1H), 3.15 (s, 1H). ^{13}C{^{1}H} NMR (101 MHz, DMSO-d_6) δ 135.0, 134.64, 134.63, 133.8, 133.0, 131.5, 131.4, 130.5, 127.4, 123.4, 46.2, 20.7. HRMS (ESI) m/z: $[M+H]^+$Calcd for $C_{12}H_{11}N_6$ $[M+H]^+$ 239.1040; Found 239.1040.

*8-Phenyl-9H-benzo[f]bis([1,2,3]triazolo)[1,5-a:1',5'-d][1,4]diazepine (**21**)*

tert-Butyl (1-diazo-2-oxopropyl)sulfonyl(phenyl)carbamate (**1**, 300 mg, 0.88 mmol, 1.5 equiv), *o*-azidobenzaldehyde (**3a**, 87 mg, 0.59 mmol, 1.0 equiv), molecular sieves (200 mg), and methanol (8 mL) were placed in screw-capped glass vial. The reaction mixture was stirred for 5 min. 3-Phenylprop-2-yn-1-amine **20** (128 mg, purity = 90 mass %, 0.88 mmol, 1.5 equiv) was added to the mixture. The resulting suspension was stirred for 48 h at room temperature. The mixture was adsorbed onto silica and chromatographed with a hexanes/ethyl acetate system using gradient elution (equilibration - 100:0, gradient 95:5 → 40:60). The solid obtained was triturated with hexane/diethyl ether = 1:1 (2 mL) twice. The residue was dried in vacuo to afford benzobistriazolodiazepine **21**.

Yield: 100 mg (57%). Beige powder; mp 210-211 °C. ^{1}H NMR (400 MHz, DMSO-d_6) δ 8.30 (s, 1H), 8.21 (dd, J = 8.1, 1.4 Hz, 1H), 7.98 (dd, J = 7.7, 1.6 Hz, 1H), 7.86 – 7.73 (m, 4H), 7.65 – 7.58 (m, 2H), 7.56 – 7.50 (m, 1H), 6.06 (s, 2H). ^{13}C{^{1}H} NMR (101 MHz, DMSO-d_6) δ 143.8, 134.1, 133.6, 132.7, 131.3, 130.0, 129.9, 129.6, 129.3, 129.2, 128.8, 127.6, 124.2, 118.1, 41.0. HRMS (ESI) m/z: $[M+H]^+$Calcd for $C_{17}H_{13}N_6$ $[M+H]^+$ 301.1196; Found 301.1200

IV. Biological assay

Cell culture

NCI-H460 and A549 lung carcinoma cell lines and were purchased from the ATCC. Cancer cells were maintained in Advanced RPMI-1640 (Gibco, UK) supplemented with 5% fetal bovine serum (FBS, Gibco, UK), penicillin (100 UI mL^{-1}), streptomycin (100 µg mL^{-1}) and GlutaMax (2 mM, Gibco, UK). All cells line cultivation under a humidified atmosphere of 95% air/5% CO_2 at 37 °C. Subconfluent monolayers, in the log growth phase, were harvested by a brief treatment with TrypLE Express solution (Gibco, UK) in phosphate buffered saline (PBS, Capricorn Scientific, Germany) and washed three times in serum-free PBS. The number of viable cells was determined by trypan blue exclusion.

Antiproliferative assay

The effects of the synthesized compounds on cell viability were determined using the MTT colorimetric test. All examined cells were diluted with the growth medium to 3.5×10^4 cells per mL and the aliquots (7×10^3 cells per 200 µL) were placed in individual wells in 96-multiplates (Eppendorf, Germany) and incubated for 24 h. The next day the cells were then treated with synthesized compounds separately at the final concentration 250 µM and incubated for 72 h at 37 °C in 5% CO_2 atmosphere. After incubation, the cells were then treated with 40 µL MTT solution (3-(4,5-dimethylthiazol-2-yl)-2,5-diphenyltetrazolium bromide, 5 mg mL^{-1} in PBS) and incubated 4 h. After an additional 4h incubation, the medium with MTT was removed and DMSO (150 µL) was added to dissolve the crystals formazan. The plates were shaken for 10 min. The optical density of each well was determined at 560 nm using a microplate reader GloMax Multi+ (Promega, USA). Each of the tested compounds was evaluated for cytotoxicity in three separate experiments.

V. NMR spectra

For NMR spectra not to contain peaks of Et_2O, DMSO was added to an analytical amounts of compounds **5d,f–i**. Et_2O was removed in vacuo. DMSO was removed using lyophilizer.

Copies of ^{1}H (400.13 MHz, DMSO-*d*$_6$) and ^{13}C{^{1}H} (100.61 MHz, DMSO-*d*$_6$) of **5a**

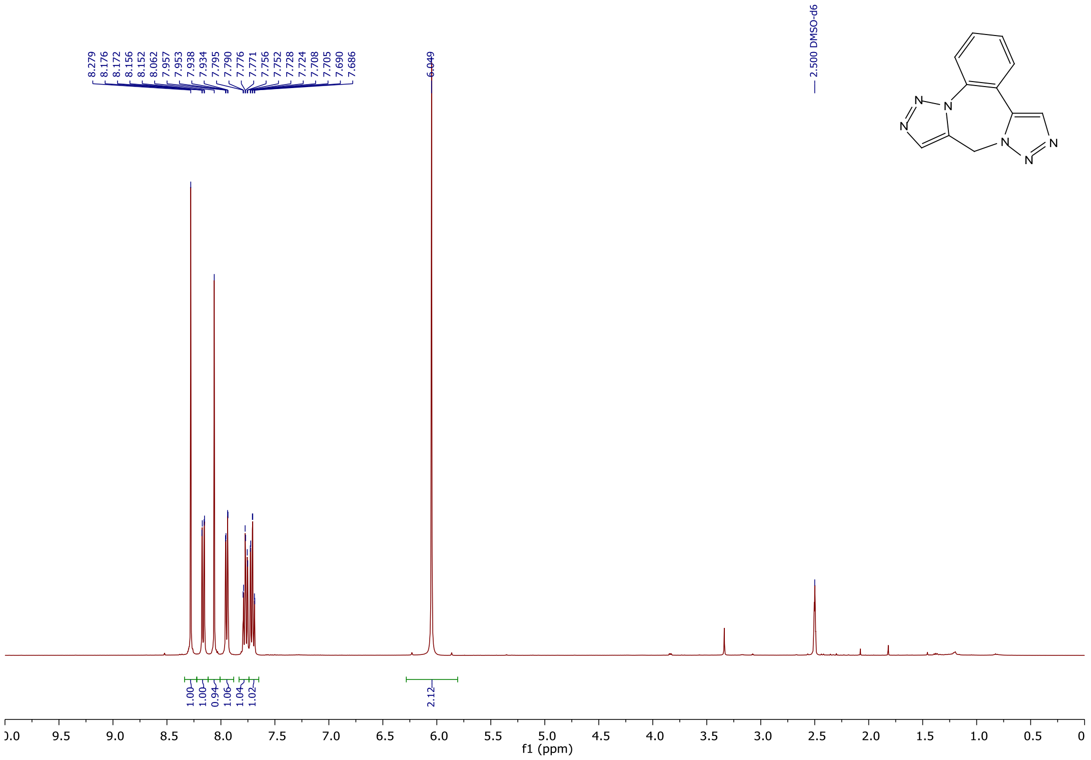

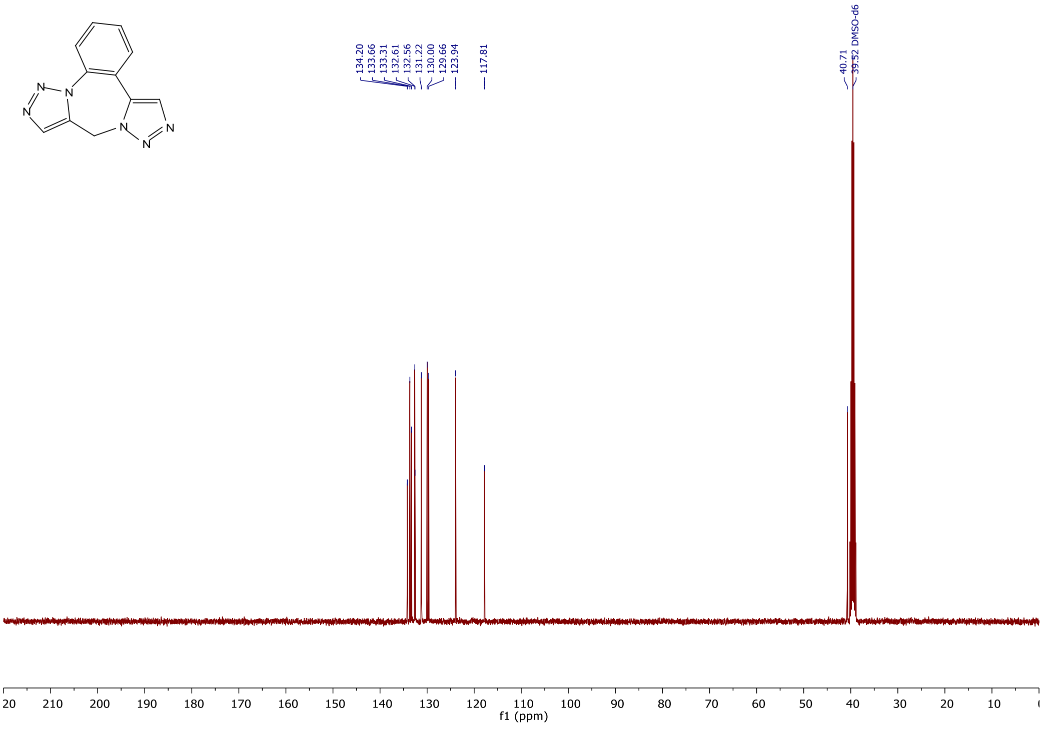

Copies of ^{1}H (400.13 MHz, DMSO-d_6) and $^{13}C\{^{1}H\}$ (100.61 MHz, DMSO-d_6) of **5b**

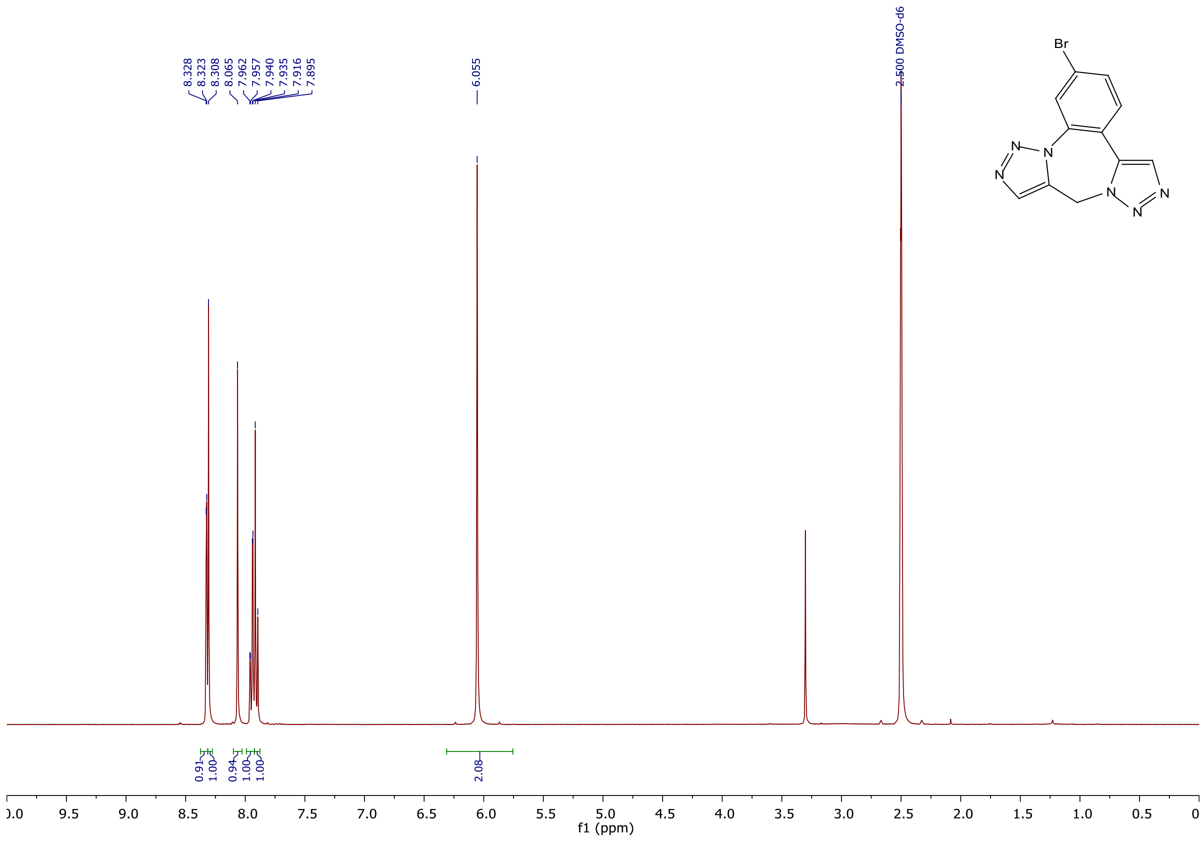

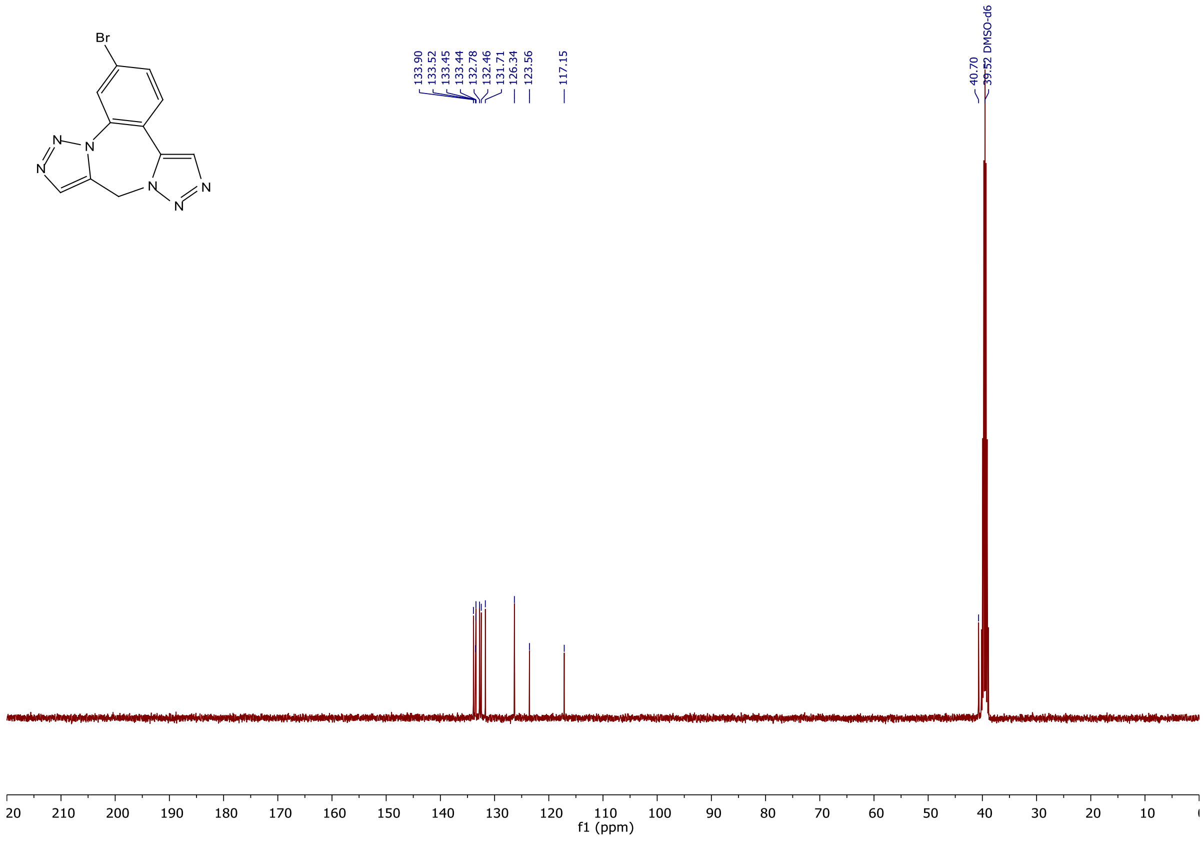

Copies of ^{1}H (400.13 MHz, DMSO-d_6) and $^{13}C\{^{1}H\}$ (100.61 MHz, DMSO-d_6) of **5c**

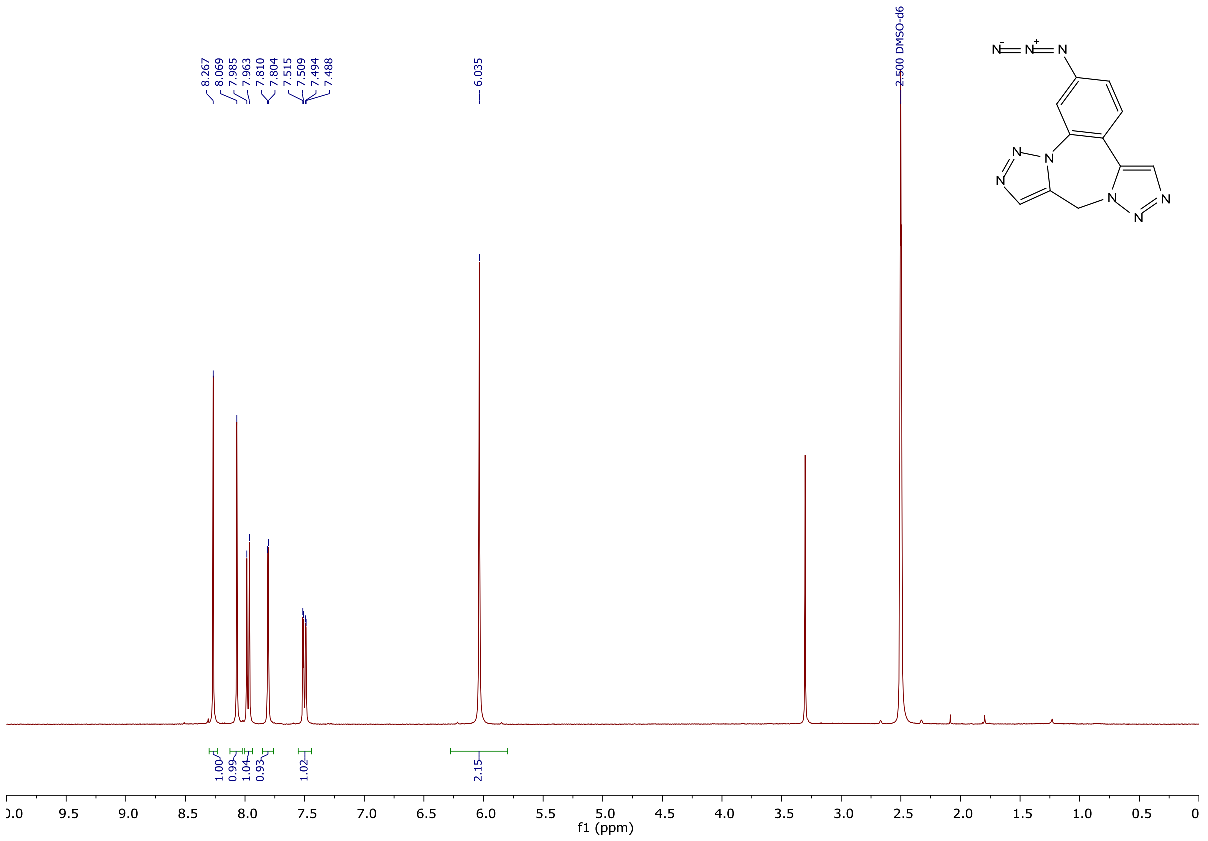

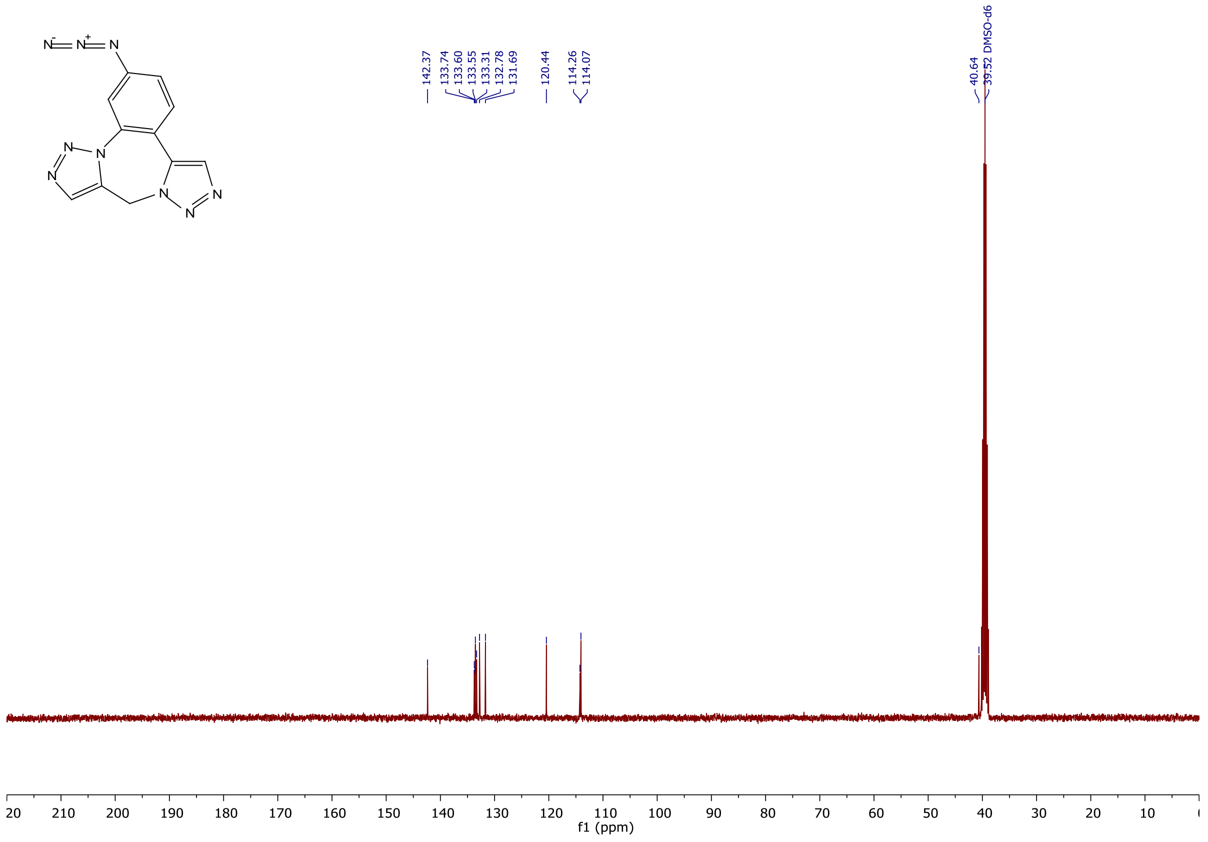

Copies of ^{1}H (400.13 MHz, DMSO-d_6) and ^{13}C{^{1}H} (100.61 MHz, DMSO-d_6) of **5d**

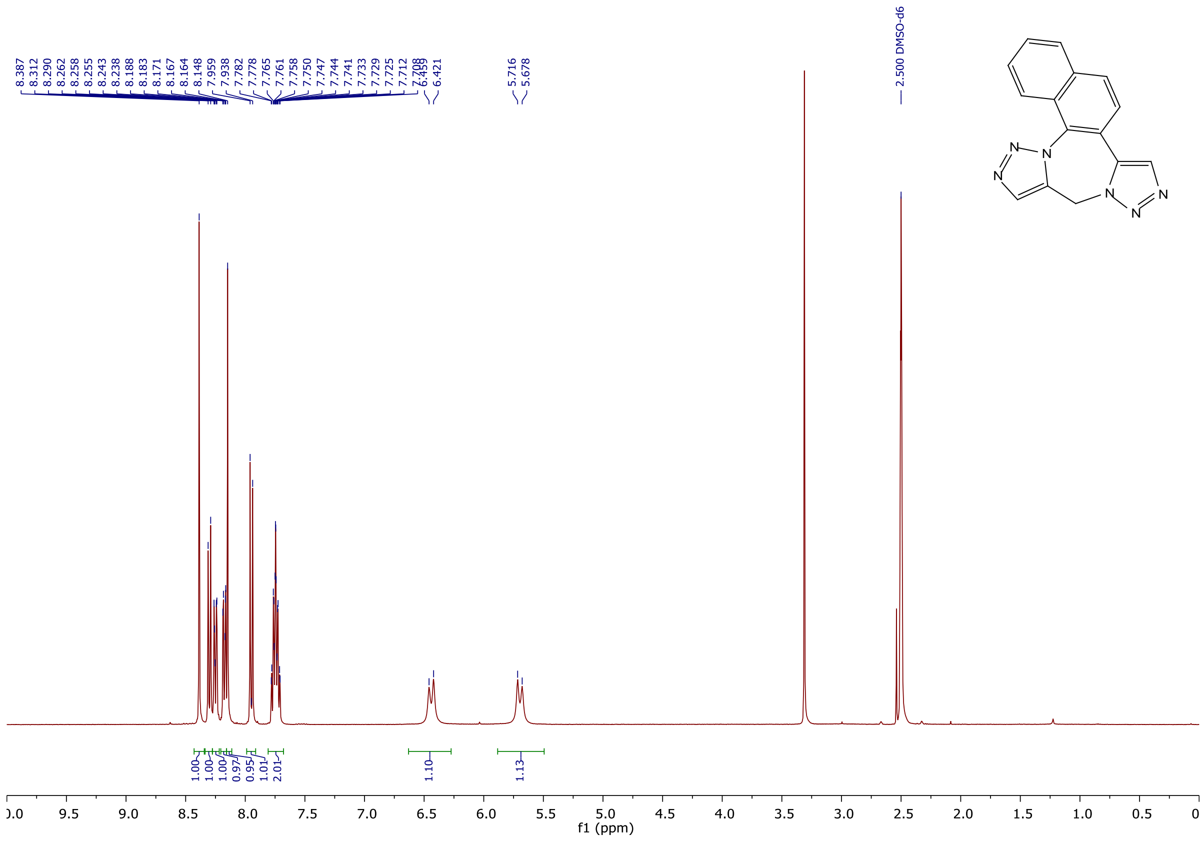

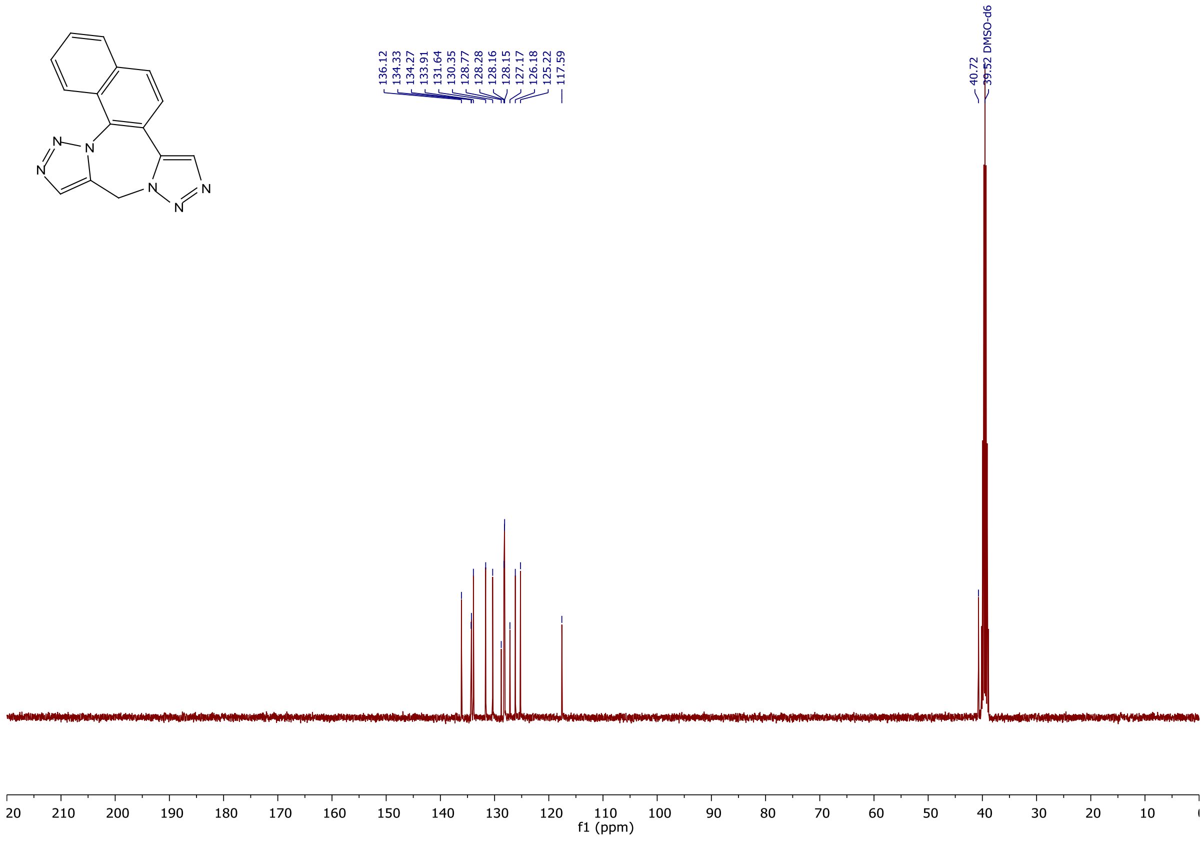

Copies of ^{1}H (400.13 MHz, DMSO-d_6) and $^{13}C\{^{1}H\}$ (100.61 MHz, DMSO-d_6) of **5e**

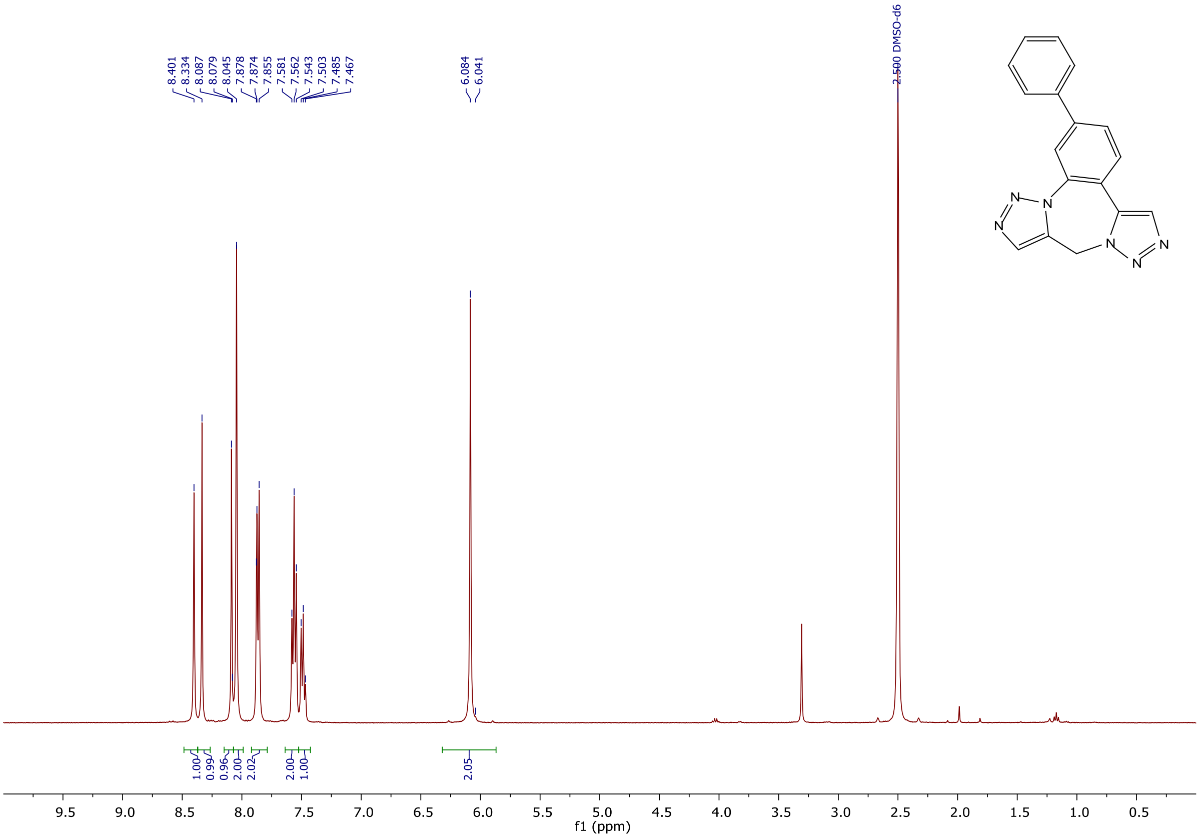

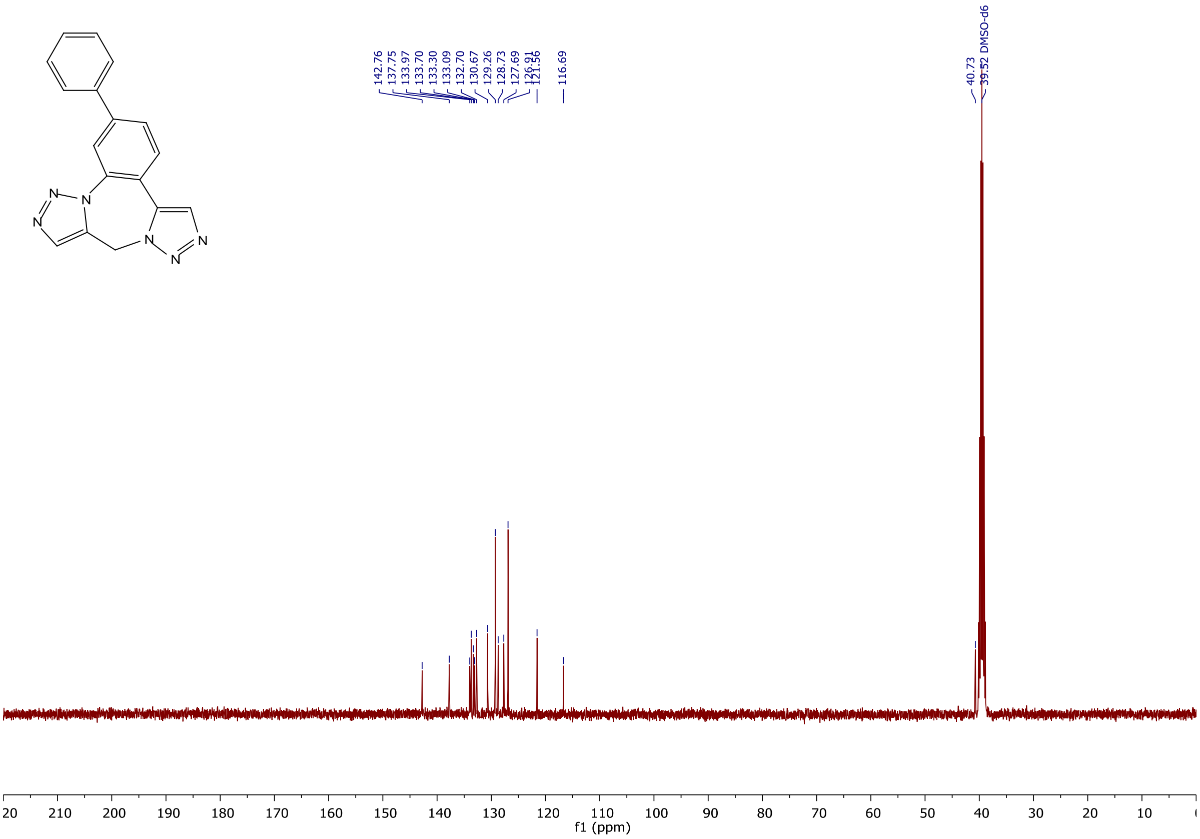

Copies of ^{1}H (400.13 MHz, DMSO-d_6) and $^{13}C\{^{1}H\}$ (100.61 MHz, DMSO-d_6) of **5f**

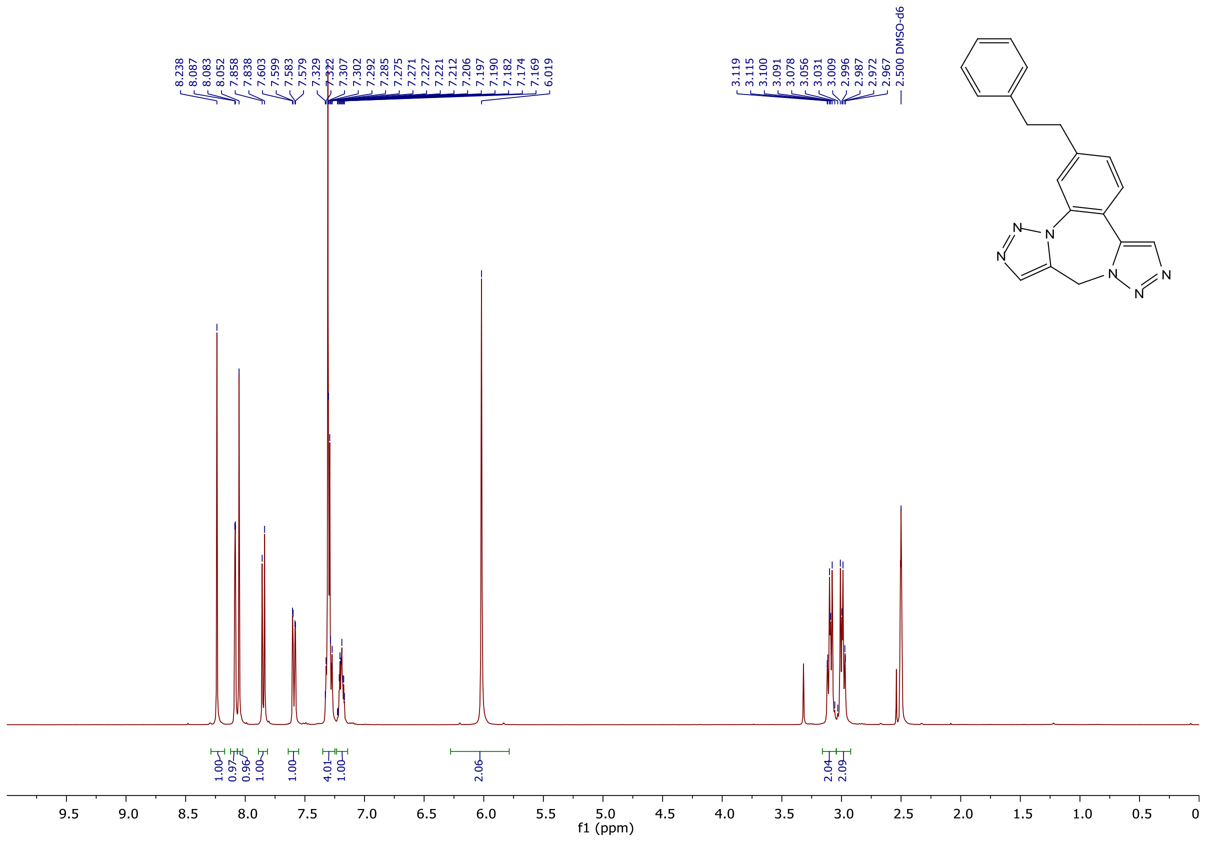

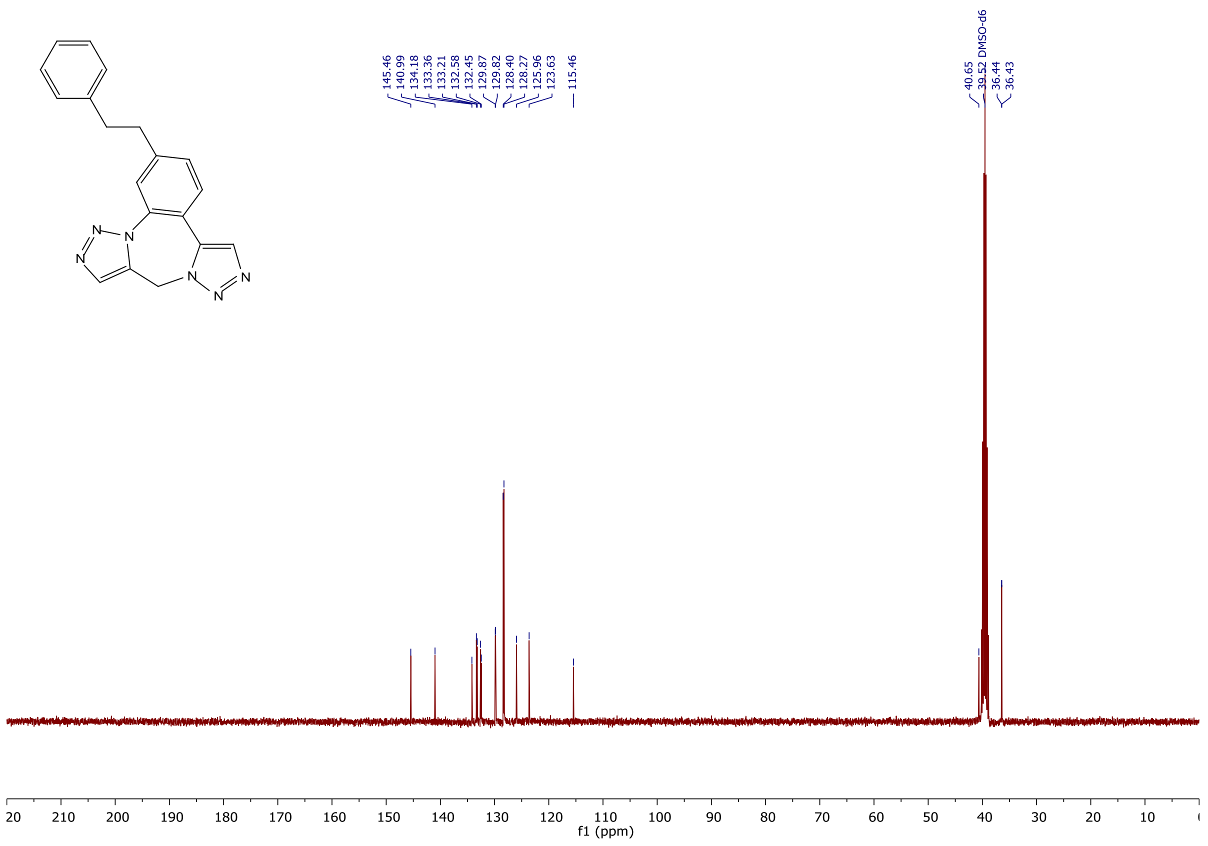

Copies of ^{1}H (400.13 MHz, DMSO-d_6) and $^{13}C\{^{1}H\}$ (100.61 MHz, DMSO-d_6) of **5g**

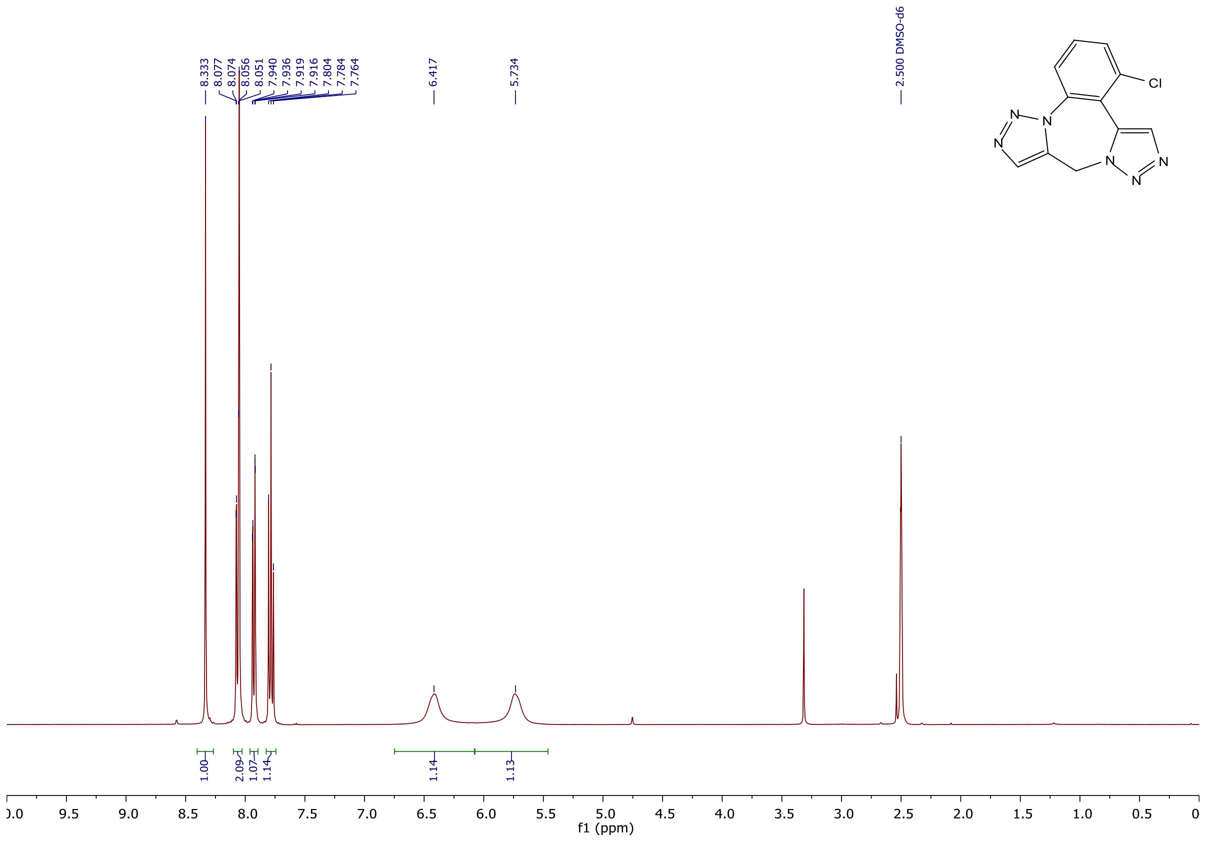

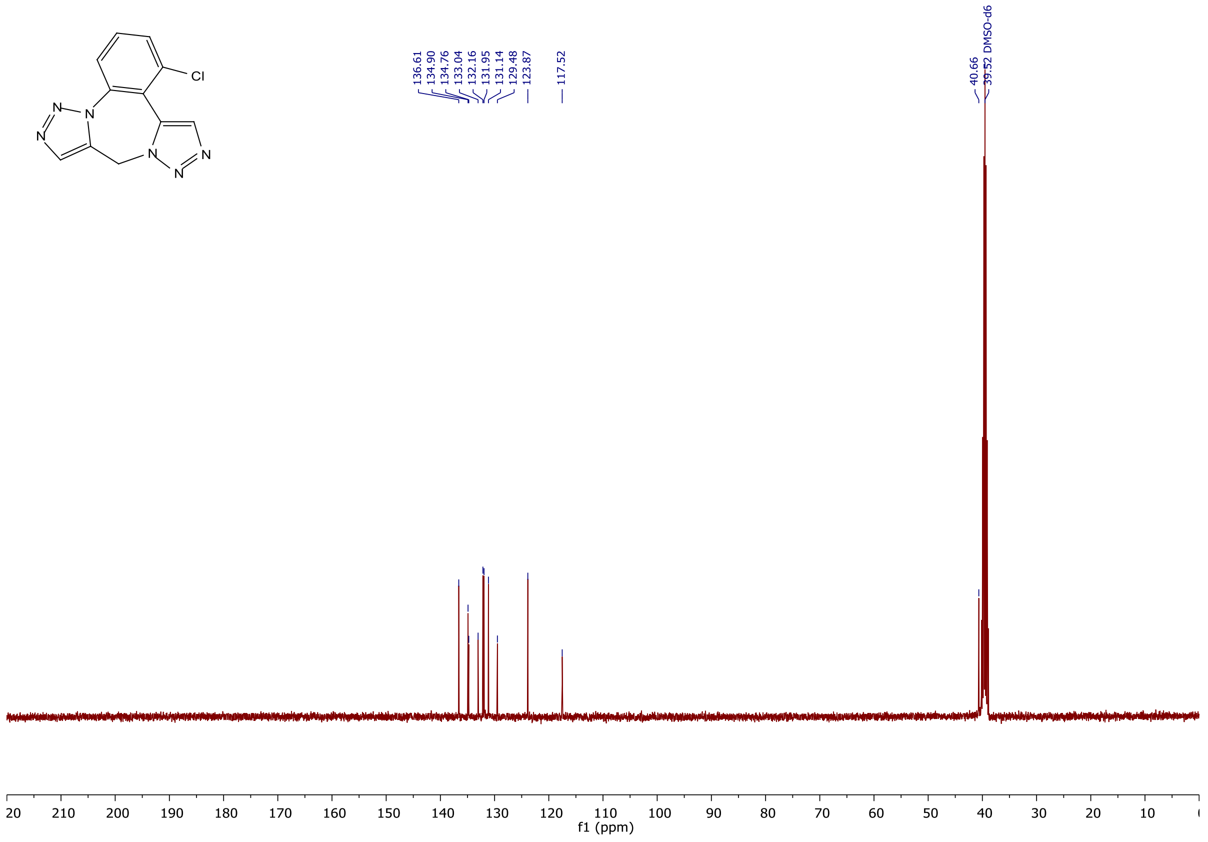

Copies of ^{1}H (400.13 MHz, DMSO-d_6) and ^{13}C{^{1}H} (100.61 MHz, DMSO-d_6) of **5h**

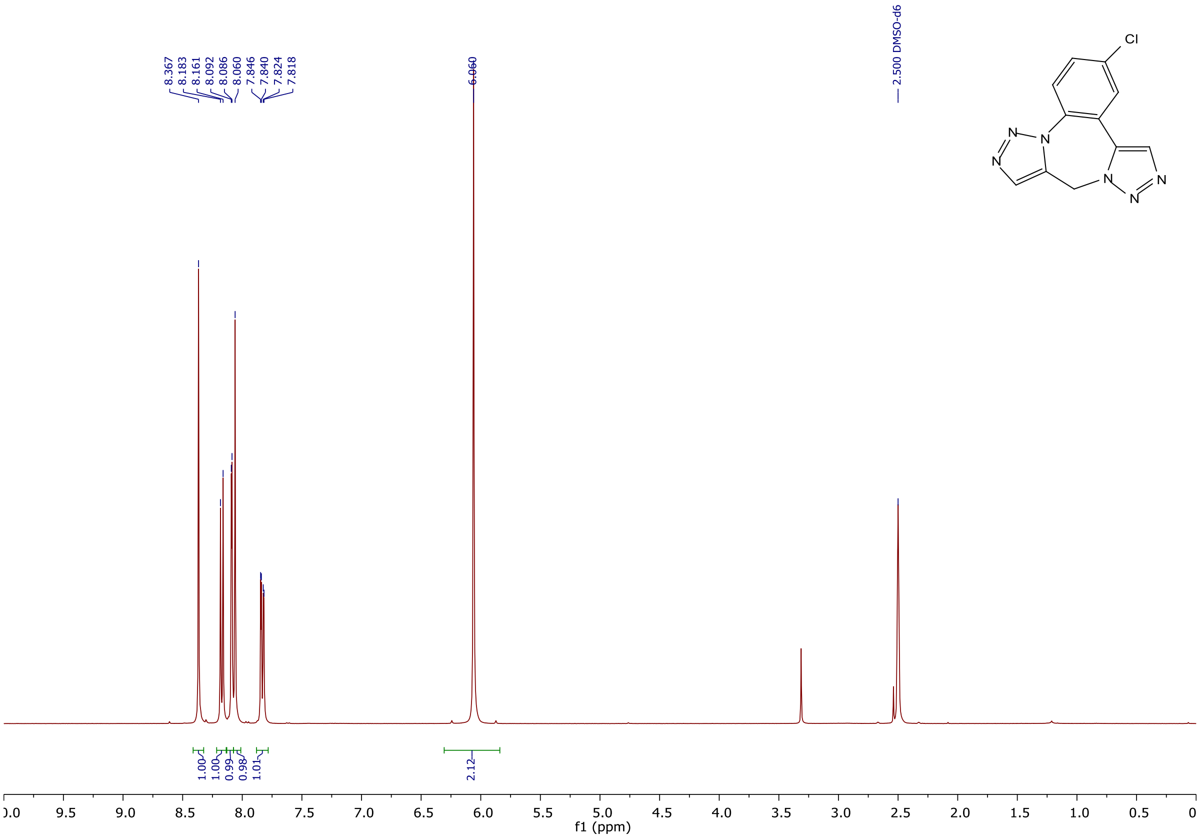

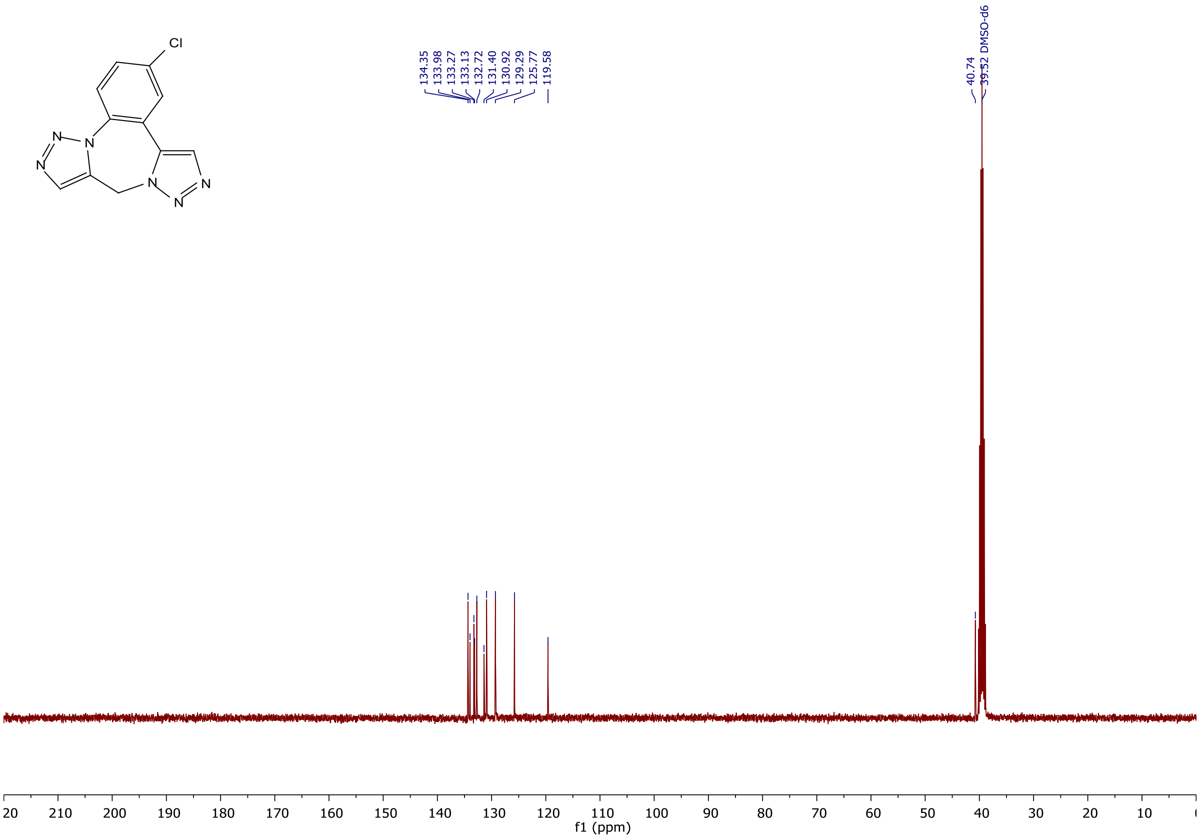

Copies of ^{1}H (400.13 MHz, DMSO-d_6) and $^{13}C\{^{1}H\}$ (125.73 MHz, DMSO-d_6) of **5i**

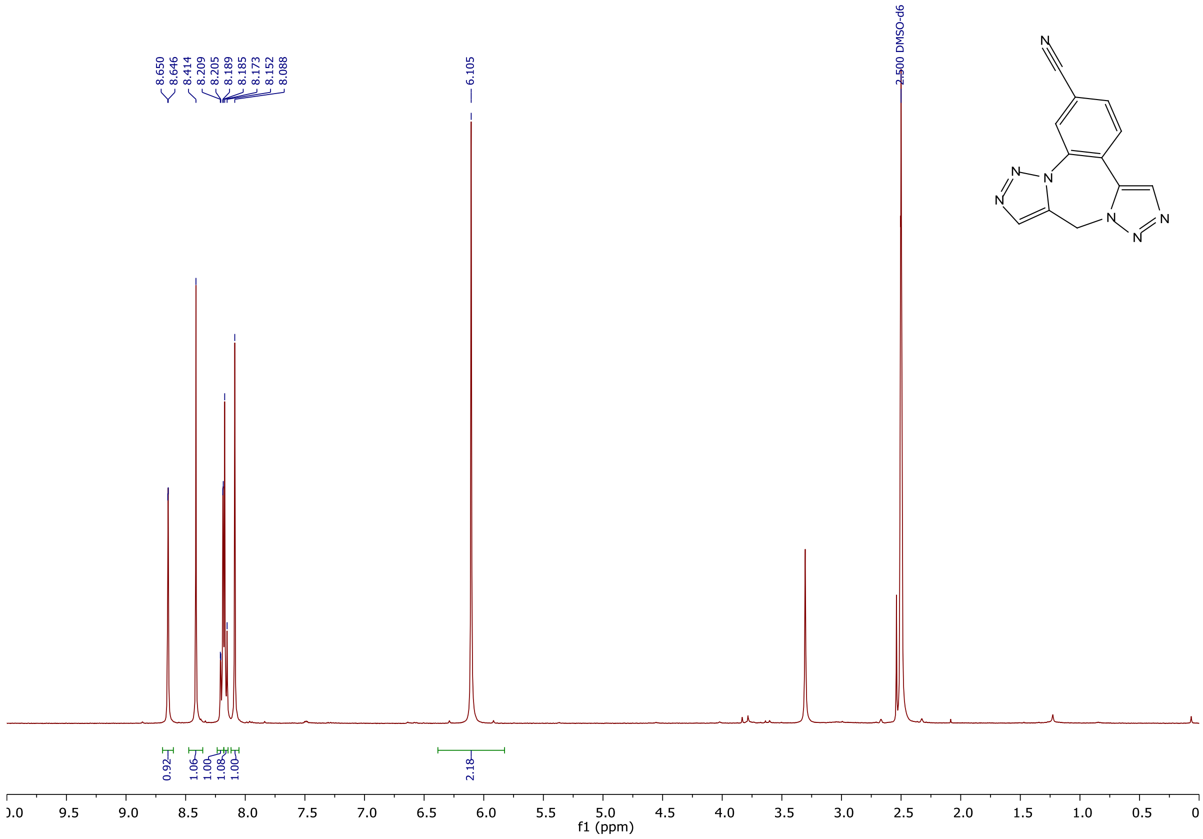

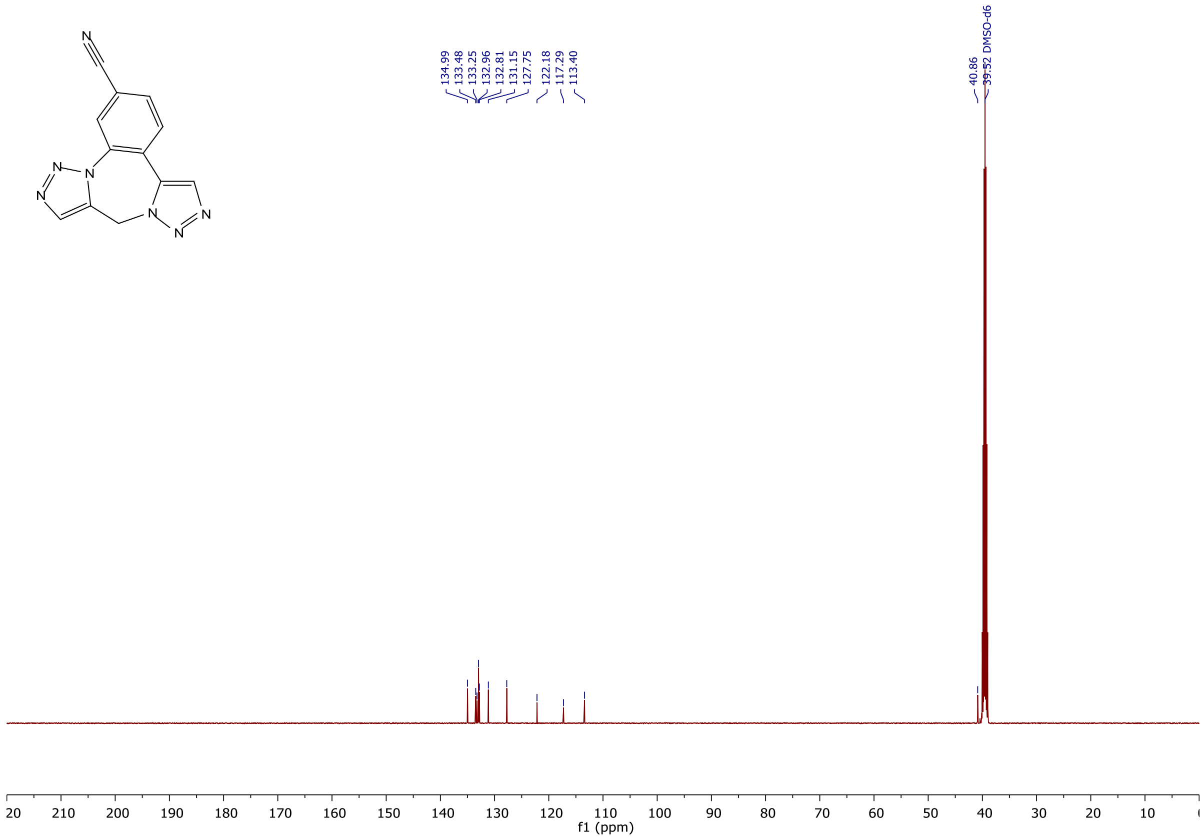

Copies of ^{1}H (400.13 MHz, DMSO-d_6) and ^{13}C{^{1}H} (100.61 MHz, DMSO-d_6) of **17**

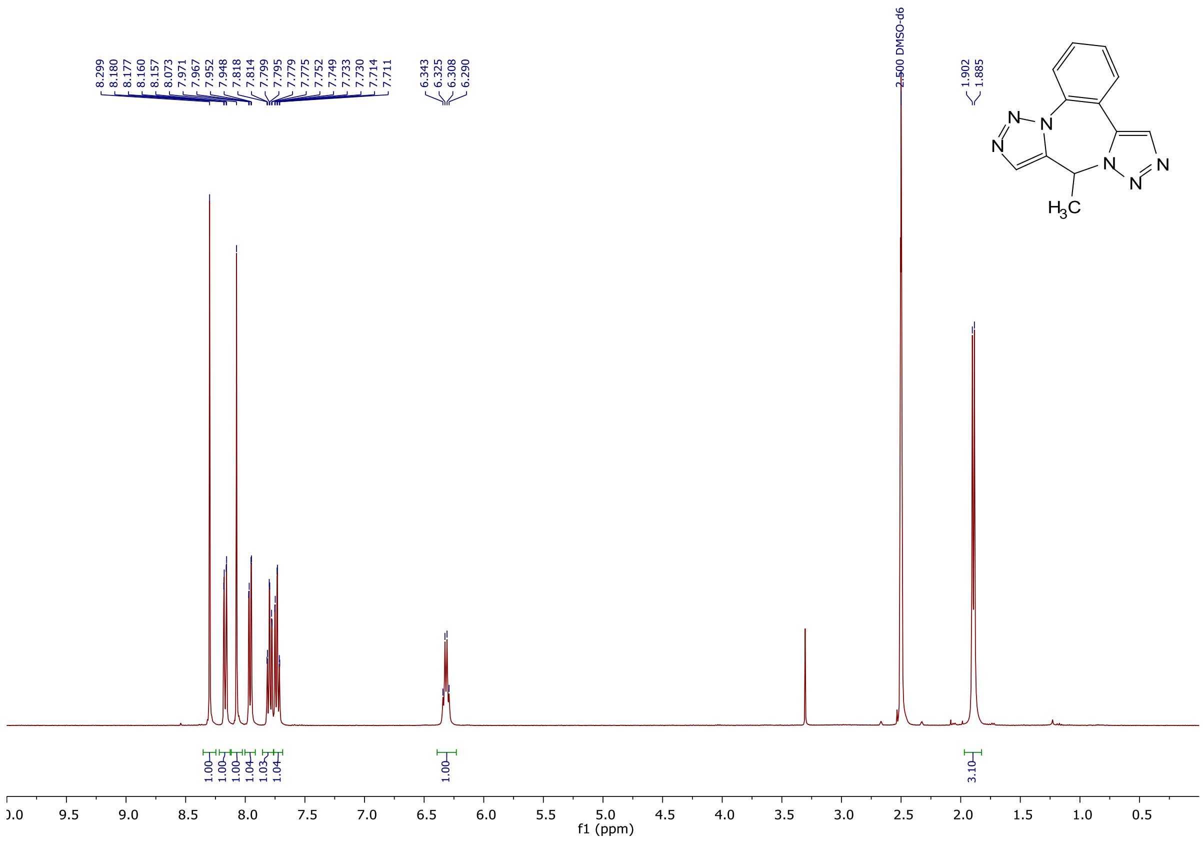

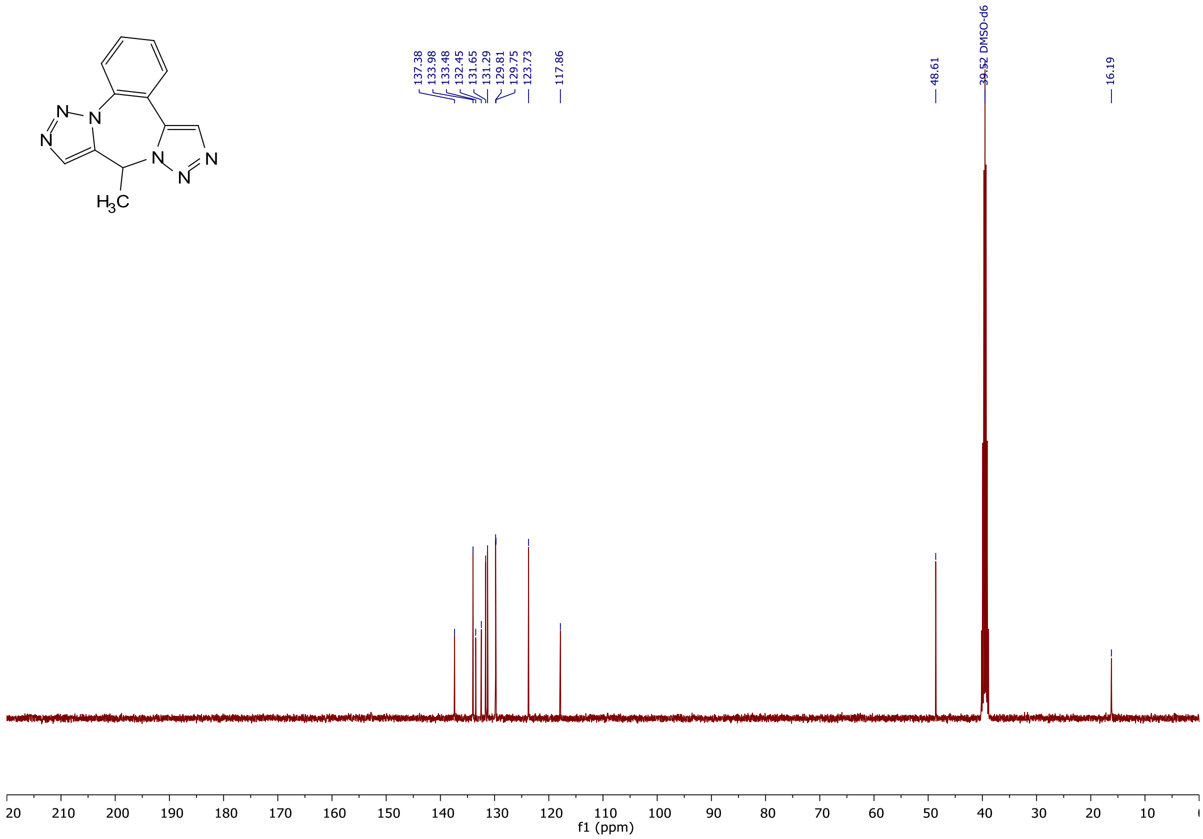

Copies of ^{1}H (400.13 MHz, DMSO-d_6) and $^{13}C\{^{1}H\}$ (100.61 MHz, DMSO-d_6) of **19**

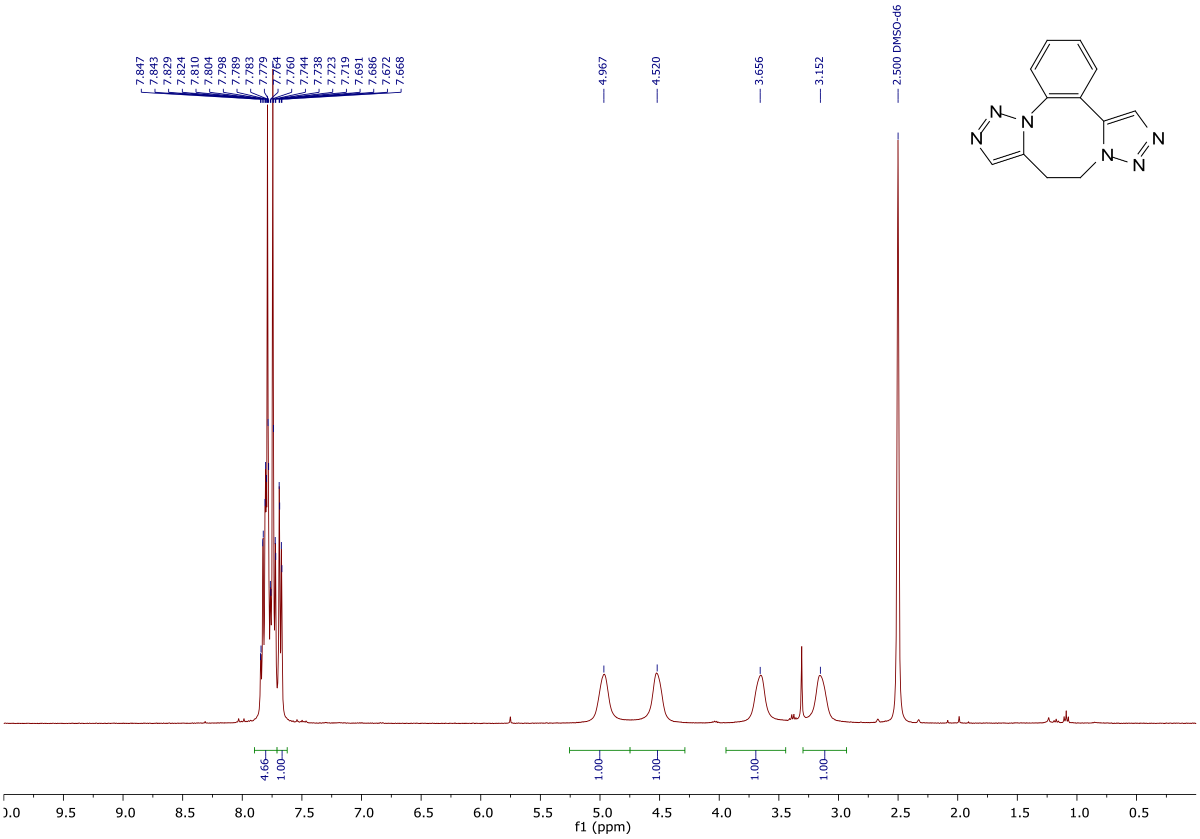

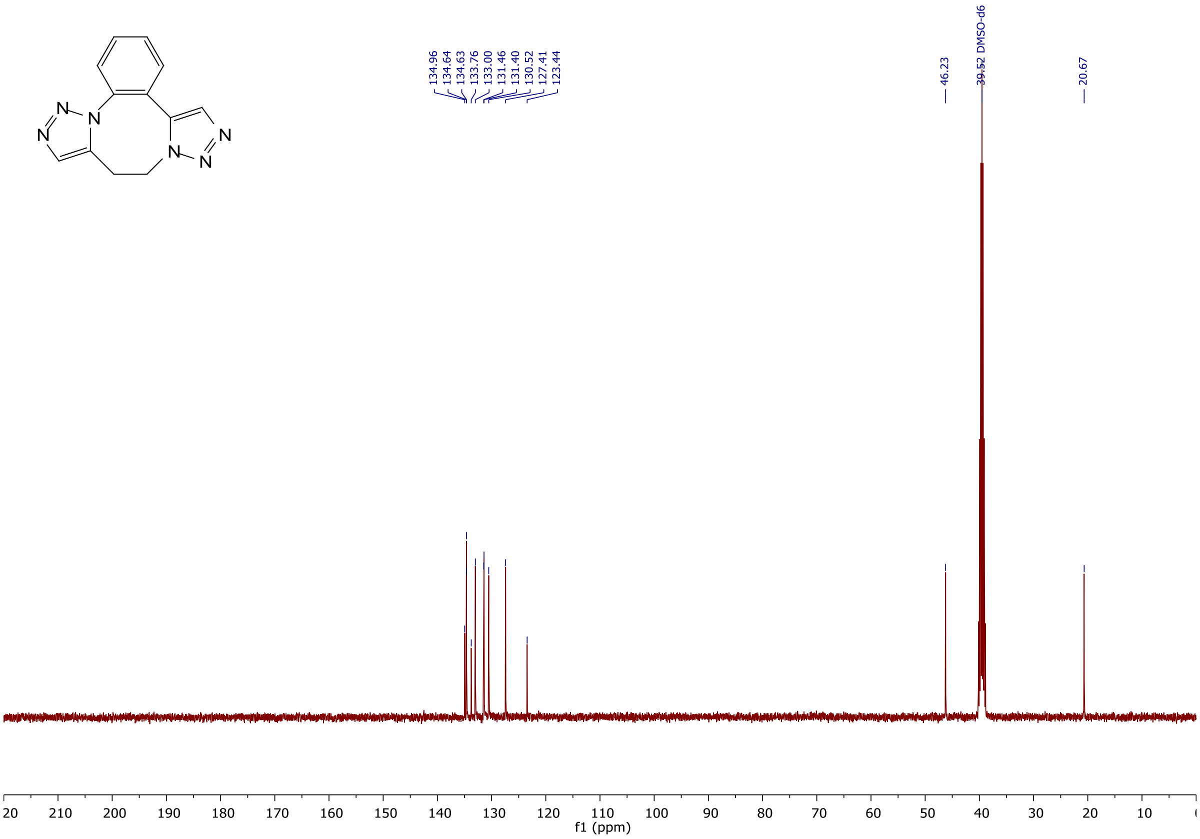

Copies of ^{1}H (400.13 MHz, DMSO-d_6) and $^{13}C\{^{1}H\}$ (100.61 MHz, DMSO-d_6) of **21**

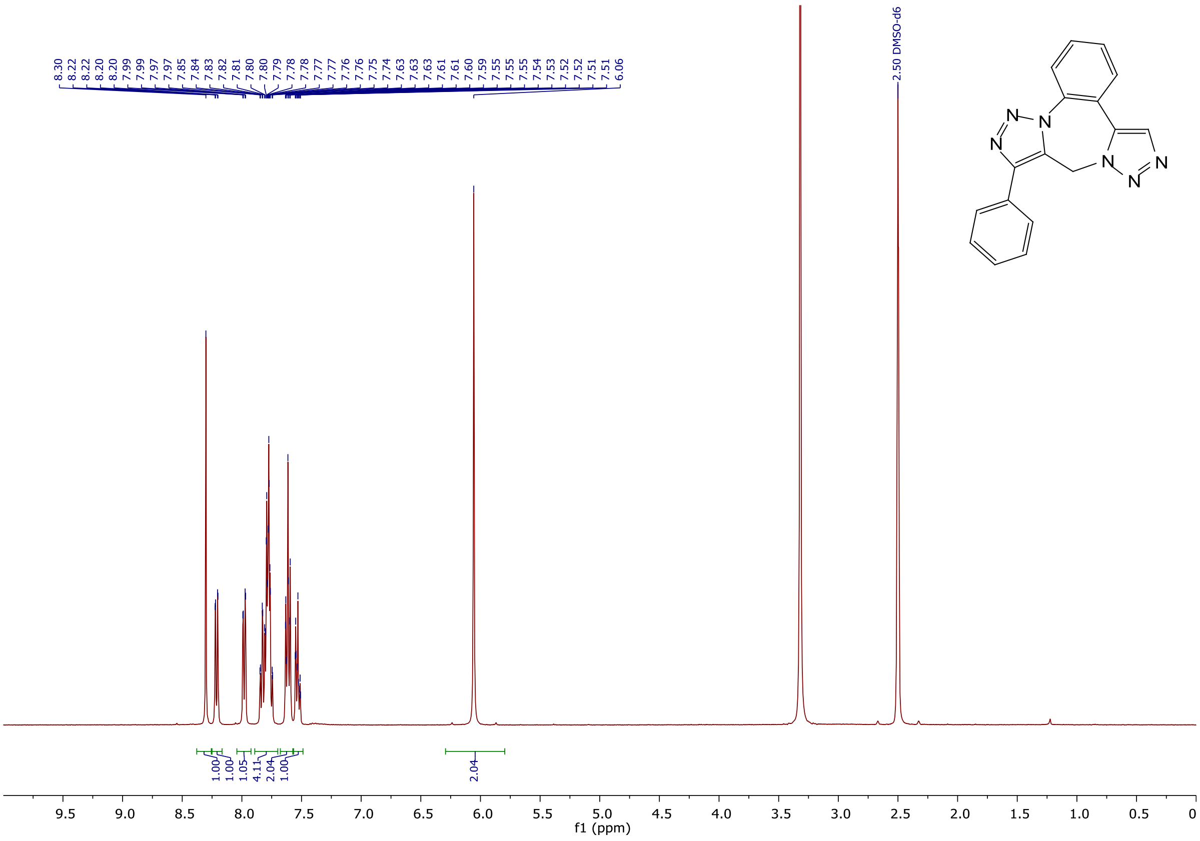

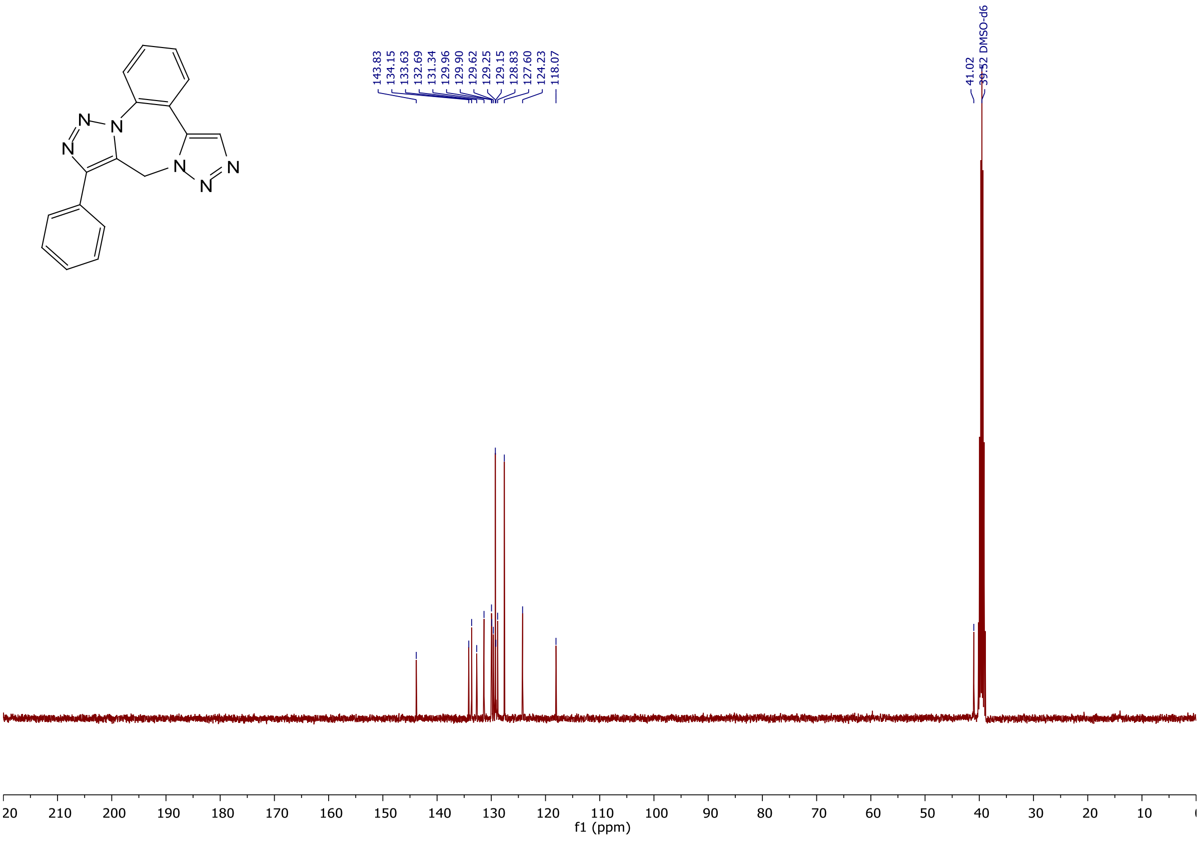

VI. X-ray crystallographic data

Crystallographic data for compounds **5a**, **19** and **21**. X-ray Single Crystal analysis was performed on RigakuXtaLAB Synergy-S diffractometer with monochromated CuKα radiation. Crystals were kept at 100 K during data collection. Using Olex2[22], the structures were solved with the SHELXT[23] structure solution program using Intrinsic Phasing and refined with the SHELXL[24] refinement package using Least Squares minimization. CCDC 2183765 **(5a)**, CCDC 2183766 **(19)** and CCDC 2183767 **(21)** contain the supplementary crystallographic data for this paper. These data can be obtained free of charge from The Cambridge Crystallographic Data Centre via https://www.ccdc.cam.ac.uk/.

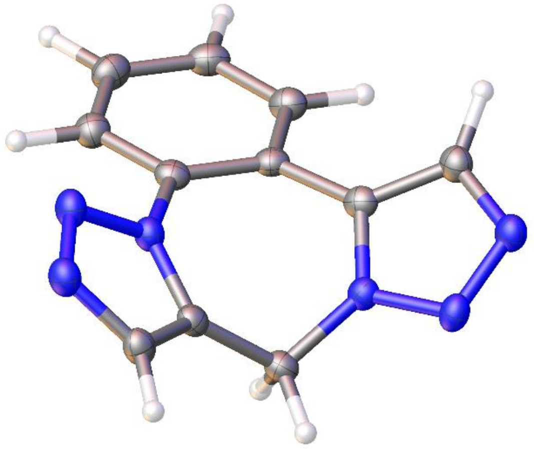

Figure S1. ORTEP representation of compound № (thermal ellipsoids are shown at 50% probability)

**Table S1.** Crystal data and structure refinement for **5a**	
CCDC	2183765
Empiricalformula	$C_{11}H_8N_6$
Formula weight	224.30
Temperature/K	100(2)
Crystal system	monoclinic
Space group	$P2_1/c$
a/Å	3.9602(2)
b/Å	14.4040(5)
c/Å	17.0972(7)
α/°	90
β/°	94.407(4)
γ/°	90
Volume/Å^3	972.39(7)
Z	4
ρ_{calc}g/cm^3	1.532
μ/mm^{-1}	0.836
F(000)	464.0
Crystal size/mm^3	0.12 × 0.1 × 0.03

Radiation	CuKα (λ = 1.54184)
2Θ range for data collection/°	8.036 to 134.992
Index ranges	-4 ≤ h ≤ 2, -16 ≤ k ≤ 17, -19 ≤ l ≤ 20
Reflectionscollected	3299
Independent reflections	1739 [R_{int} = 0.0521, R_{sigma} = 0.0568]
Data/restraints/parameters	1739/0/154
Goodness-of-fit on F^2	1.052
Final R indexes [I>=2σ (I)]	R_1 = 0.0557, wR_2 = 0.1449
Final R indexes [all data]	R_1 = 0.0738, wR_2 = 0.1603
Largest diff. peak/hole / e $Å^{-3}$	0.32/-0.30

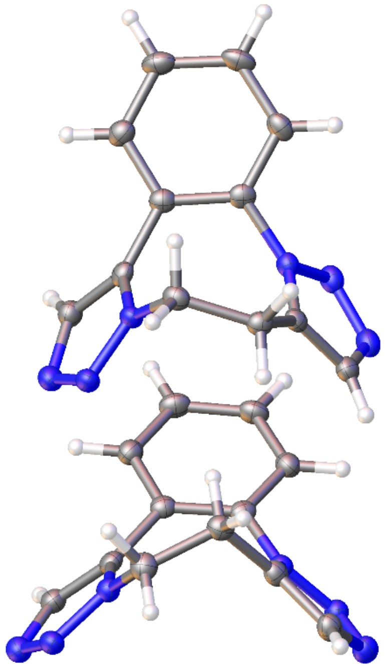

Figure S2. ORTEP representation of compound № (thermal ellipsoids are shown at 50% probability)

**Table S2.** Crystal data and structure refinement for **19**	
CCDC	2183766
Empiricalformula	$C_{12}H_{10}N_6$
Formula weight	238.26
Temperature/K	100(1)
Crystal system	orthorhombic
Space group	$P2_12_12_1$
a/Å	8.7035(2)
b/Å	9.1556(3)
c/Å	27.0191(7)
α/°	90
β/°	90
γ/°	90
Volume/$Å^3$	2153.04(10)

Z	8
$\rho_{calc}g/cm^3$	1.470
μ/mm^{-1}	0.789
F(000)	992.0
Crystal size/mm^3	0.16 × 0.11 × 0.04
Radiation	Cu Kα (λ = 1.54184)
2Θ range for data collection/°	6.542 to 134.98
Index ranges	-9 ≤ h ≤ 10, -9 ≤ k ≤ 10, -23 ≤ l ≤ 32
Reflectionscollected	8190
Independent reflections	3673 [R_{int} = 0.0361, R_{sigma} = 0.0445]
Data/restraints/parameters	3673/0/325
Goodness-of-fit on F^2	1.008
Final R indexes [I>=2σ (I)]	R_1 = 0.0390, wR_2 = 0.0994
Final R indexes [all data]	R_1 = 0.0410, wR_2 = 0.1005
Largest diff. peak/hole / e $Å^{-3}$	0.28/-0.29

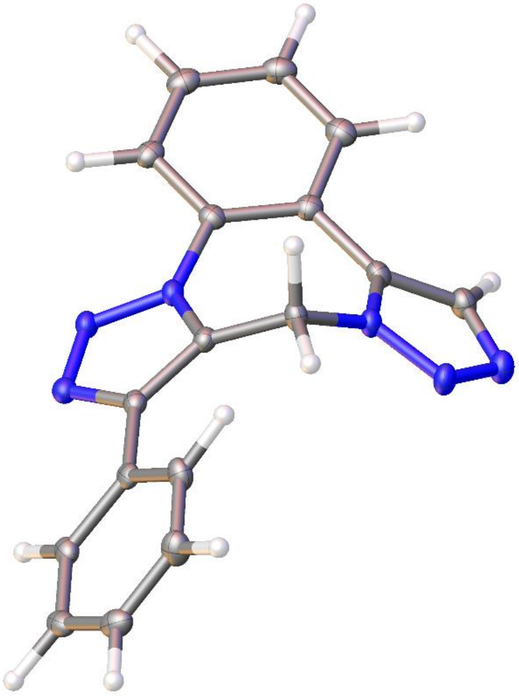

Figure S3. ORTEP representation of compound № (thermal ellipsoids are shown at 50% probability)

**Table S3.** Crystal data and structure refinement for **21**	
CCDC	2183767
Empiricalformula	$C_{17}H_{12}N_6$
Formula weight	300.33
Temperature/K	100(2)
Crystal system	monoclinic
Space group	C2/c
a/Å	26.4589(4)

b/Å	5.79540(10)
c/Å	19.7117(3)
α/°	90
β/°	110.118(2)
γ/°	90
Volume/Å^3	2838.17(9)
Z	8
ρ_{calc}g/cm^3	1.406
μ/mm^{-1}	0.726
F(000)	1248.0
Crystal size/mm^3	0.18 × 0.14 × 0.05
Radiation	Cu Kα (λ = 1.54184)
2Θ range for data collection/°	7.116 to 134.994
Index ranges	-31 ≤ h ≤ 31, -6 ≤ k ≤ 6, -23 ≤ l ≤ 22
Reflectionscollected	17179
Independent reflections	2548 [R_{int} = 0.0586, R_{sigma} = 0.0352]
Data/restraints/parameters	2548/0/208
Goodness-of-fit on F^2	1.047
Final R indexes [I>=2σ (I)]	R_1 = 0.0388, wR_2 = 0.1011
Final R indexes [all data]	R_1 = 0.0439, wR_2 = 0.1062
Largest diff. peak/hole / e Å^{-3}	0.19/-0.29

VII. References

1. Bubyrev, A.; Malkova, K.; Kantin, G.; Dar'in, D.; Krasavin, M., Metal-Free Synthesis of 1,5-Disubstituted 1,2,3-Triazoles. *The Journal of Organic Chemistry* **2021,** *86* (23), 17516-17522.

2. Del Vecchio, A.; Talbot, A.; Caillé, F.; Chevalier, A.; Sallustrau, A.; Loreau, O.; Destro, G.; Taran, F.; Audisio, D., Carbon isotope labeling of carbamates by late-stage [11C], [13C] and [14C]carbon dioxide incorporation. *Chemical Communications* **2020,** *56* (78), 11677-11680.

3. Trost, B. M.; Osipov, M.; Krüger, S.; Zhang, Y., A catalytic asymmetric total synthesis of (−)-perophoramidine. *Chemical Science* **2015,** *6* (1), 349-353.

4. Stokes, B. J.; Vogel, C. V.; Urnezis, L. K.; Pan, M.; Driver, T. G., Intramolecular Fe(II)-Catalyzed N−O or N−N Bond Formation from Aryl Azides. *Organic Letters* **2010,** *12* (12), 2884-2887.

5. Bühler, S.; Lagoja, I.; Giegrich, H.; Stengele, K.-P.; Pfleiderer, W., New Types of Very Efficient Photolabile Protecting Groups Based upon the [2-(2-Nitrophenyl)propoxy]carbonyl (NPPOC) Moiety. *Helvetica Chimica Acta* **2004,** *87* (3), 620-659.

6. Zhang, C.; Zhang, L.; Sun, C.; Sun, W.; Liu, X., BN-Phenanthrenes: Synthesis, Reactivity, and Optical Properties. *Organic Letters* **2019,** *21* (10), 3476-3480.

7. Stokes, B. J.; Liu, S.; Driver, T. G., Rh2(II)-Catalyzed Nitro-Group Migration Reactions: Selective Synthesis of 3-Nitroindoles from β-Nitro Styryl Azides. *Journal of the American Chemical Society* **2011,** *133* (13), 4702-4705.

8. Nielsen, S., Feldbæk; Schønning, K.; Kromann, H.; Boesen, T.; Larsen, M. Diamino-functional chalcones. WO2003097575, 27.11.2003.

9. Tatani, K.; Nonaka, Y.; Kikuchi, N. Purinenucleoside derivative modified in 8-position and medicinal use thereof. 23.03.2006.

10. Hamsmann, M., Martin; Hashmi, A. S. K.; Romero-Nieto, C.; López-Andarias, A.; Rettenmeier, E.; Egler-Lucas, C. Novel compounds exhibiting photophysical properties upon formation of lewis acid-base adducts using non-chelating boranes, method for producing the same and devices including the same. WO2017016653, 02.02.2017.

11. Dilger, A. K.; Pabbisetty, K. B.; Corte, J. R.; De Lucca, I.; Fang, T.; Yang, W.; Pinto, D. J. P.; Wang, Y.; Zhu, Y.; Mathur, A.; Li, J.; Hou, X.; Smith, D.; Sun, D.; Zhang, H.; Krishnananthan, S.; Wu, D.-R.; Myers, J. E.; Sheriff, S.; Rossi, K. A.; Chacko, S.; Zheng, J. J.; Galella, M. A.; Ziemba, T.; Dierks, E. A.; Bozarth, J. M.; Wu, Y.; Crain, E.; Wong, P. C.; Luettgen, J. M.; Wexler, R. R.; Ewing, W. R., Discovery of Milvexian, a High-Affinity, Orally Bioavailable Inhibitor of Factor XIa in Clinical Studies for Antithrombotic Therapy. *Journal of Medicinal Chemistry* **2022,** *65* (3), 1770-1785.

12. Schirmer, H.; Rubenbauer, P.; Keil, B.; Olenik, B. Method for producing (4s)-4-[4-cyano-2-(methylsulfonyl)phenyl]-3,6-dimethyl-2-oxo-1-[3-(trifluoromethyl)phenyl]-1,2,3,4-tetrahydro pyrimidine-5-carbonitrile. WO2016146607, 22.09.2016.

13. Olsen, J.; Seiler, P.; Wagner, B.; Fischer, H.; Tschopp, T.; Obst-Sander, U.; Banner, D. W.; Kansy, M.; Müller, K.; Diederich, F., A fluorine scan of the phenylamidinium needle of tricyclic thrombin inhibitors: effects of fluorine substitution on pKa and binding affinity and evidence for intermolecular C–F⋯CN interactions. *Organic & Biomolecular Chemistry* **2004,** *2* (9), 1339-1352.

14. Abaev, V. T.; Plieva, A. T.; Chalikidi, P. N.; Uchuskin, M. G.; Trushkov, I. V.; Butin, A. V., A Simple Route to Polysubstituted Indoles Exploiting Azide Induced Furan Ring Opening. *Organic Letters* **2014,** *16* (16), 4150-4153.

15. Woo-Jin, C.; Jalani, H. B.; Jeong, J.-H., Synthesis of selenopyrano[2,3-b]indol-4(9H)-ones and their C-H arylation. *Journal of Heterocyclic Chemistry* **2022**.

16. Becher, J.; Pluta, K.; Krake, N.; Brondum, K.; Christensen, N. J.; Vinader, M. V., Syntheses of o-Aminohetarenecarbaldehydes via Azides. *Synthesis* **1989,***1989*, 530-533.

17. Ibrahim, N. M.; Yosef, H. A. A.; Mahran, M. R. H., Preparation and Reactions of 2-azidoquinoline-3-carboxaldehyde with Primary Amines and Active Methylene Compounds. *Journal of Chemical Research* **2009,***2009*, 220-224.

18. Uredi, D.; Motati, D. R.; Watkins, E. B., A Unified Strategy for the Synthesis of β-Carbolines, γ-Carbolines, and Other Fused Azaheteroaromatics under Mild, Metal-Free Conditions. *Organic Letters* **2018,***20* (20), 6336-6339.

19. Song, H.; Liu, Y.; Wang, Q., Cascade Electrophilic Iodocyclization: Efficient Preparation of 4-Iodomethyl Substituted Tetrahydro-β-carbolines and Formal Synthesis of Oxopropaline G. *Organic Letters* **2013,***15* (13), 3274-3277.

20. Karibe, Y.; Kusama, H.; Iwasawa, N., Chromium(0)-Catalyzed Tandem Cyclization of α,β-Unsaturated Thioimidates Containing an Enyne Moiety. *Angewandte Chemie International Edition* **2012,***51* (25), 6214-6218.

21. Hess, W.; Burton, J. W., Palladium-Catalysed Cyclisation of N-Alkynyl Aminomalonates. *Chemistry – A European Journal* **2010,***16* (41), 12303-12306.

22. Sakthivel, S.; Sharma, A.; Balamurugan, R., Silver-Catalyzed Synthesis of Substituted Pyridine Derivatives from N-Propargylic α-Enamino Esters. *European Journal of Organic Chemistry* **2017,***2017* (27), 3941-3946.

23. Dolomanov, O.V., Bourhis, L.J., Gildea, R.J, Howard, J.A.K. &Puschmann, H.OLEX2: a complete structure solution, refinement and analysis program. Journal of Applied Crystallography**2009**, 42, 339-341.

24. Sheldrick, G.M. .SHELXT - Integrated space-group and crystal-structure determination. Acta Crystallographica Section A**2015,** A71, 3-8.

25. Sheldrick, G.M. .Crystal Structure Refinement with SHELXL.. Acta Crystallographica Section C **2015,**C71, 3-8.
